# Application of a Synthetic Ferredoxin‐Inspired [4Fe4S]‐Peptide Maquette as the Redox Partner for an [FeFe]‐Hydrogenase

**DOI:** 10.1002/cbic.202300250

**Published:** 2023-08-13

**Authors:** Andrea Bombana, Muralidharan Shanmugam, David Collison, Alexander J. Kibler, Graham N. Newton, Christof M. Jäger, Anna K. Croft, Simone Morra, Nicholas J. Mitchell

**Affiliations:** ^1^ School of Chemistry University of Nottingham University Park Nottingham NG7 2RD UK; ^2^ Department of Chemistry The University of Manchester Oxford Road Manchester M13 9PL UK; ^3^ The GlaxoSmithKline Carbon Neutral Labs for Sustainable Chemistry University of Nottingham Jubilee Campus Triumph Road Nottingham NG7 2TU UK; ^4^ Data Science and Modelling, Pharmaceutical Sciences R&D, AstraZeneca Gothenburg Pepparedsleden 1 431 83 Mölndal Sweden; ^5^ Department of Chemical and Environmental Engineering University of Nottingham University Park Nottingham NG7 2RD UK; ^6^ Department of Chemical Engineering, School of AACME Loughborough University Loughborough LE11 3TU UK

**Keywords:** Iron-sulfur, Peptide, Maquette, Hydrogenase, EPR

## Abstract

‘Bacterial‐type’ ferredoxins host a cubane [4Fe4S]^2+/+^ cluster that enables these proteins to mediate electron transfer and facilitate a broad range of biological processes. Peptide maquettes based on the conserved cluster‐forming motif have previously been reported and used to model the ferredoxins. Herein we explore the integration of a [4Fe4S]‐peptide maquette into a H_2_‐powered electron transport chain. While routinely formed under anaerobic conditions, we illustrate by electron paramagnetic resonance (EPR) analysis that these maquettes can be reconstituted under aerobic conditions by using photoactivated NADH to reduce the cluster at 240 K. Attempts to tune the redox properties of the iron‐sulfur cluster by introducing an Fe‐coordinating selenocysteine residue were also explored. To demonstrate the integration of these artificial metalloproteins into a semi‐synthetic electron transport chain, we utilize a ferredoxin‐inspired [4Fe4S]‐peptide maquette as the redox partner in the hydrogenase‐mediated oxidation of H_2_.

## Graphical abstract

[4Fe4S]‐peptide maquettes have been explored as effective models of FeS proteins, but they also hold untapped potential as tools for synthetic biology. Here we demonstrate the application of these maquettes beyond the modelling of electron transfer in biology by substituting the physiological redox partner of a *Clostridium acetobutylicum* [FeFe]‐hydrogenase with a synthetic ferredoxin mimic to direct this enzyme‐mediated H_2_‐oxidation process.

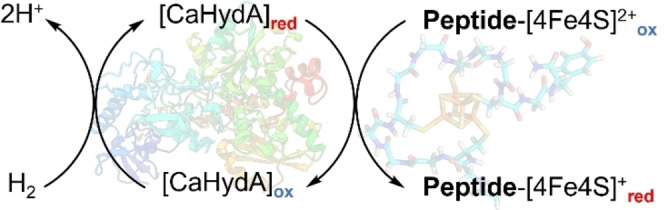

## Introduction

Iron‐sulfur (Fe−S) proteins are found ubiquitously across both prokaryotic and eukaryotic forms of life.[^1^, ^2^] Hosting several types of FeS clusters, including [2Fe2S], [3Fe4S], [4Fe3S], and [4Fe4S],[^3^, ^4^, ^5^, ^6^] these proteins act as redox mediators within metabolic, respiratory, and photosynthetic electron transport pathways, and catalyze a broad range of synthetically challenging chemistry.[^7^, ^8^, ^9^, ^10^, ^11^] Due to their central importance within bacterial metabolism, FeS proteins are postulated to have played a role in the origins of life on Earth in early prokaryotes (the iron‐sulfur‐world hypothesis).[^12^, ^13^]

The ‘bacterial‐type’ ferredoxins host a [4Fe4S]^2+/+^ cluster consisting of Fe^2+^ and Fe^3+^ ions linked in a cubic structure by four sulfide anions, and coordinated by the thiolate sidechains of four ligand cysteine (Cys) residues.^[1]^ This cluster enables them to act as redox mediators, accepting and discharging electrons, to facilitate metabolic energy transduction.^[7]^ A conserved region of their sequence, consisting of three Cys residues separated from one another by two additional variable residues (i. e., CXXCXXC), hosts the [4Fe4S] cluster, along with a fourth Cys residue located further from this motif. Spontaneous reconstitution of the cluster within recombinantly expressed ferredoxin has been extensively studied for these proteins, as has the process of biological production.[^3^, ^14^] Reconstitution of the *apo* form of the ferredoxins can be achieved under anaerobic conditions with the addition of sources of sulfide (Na_2_S), Fe(III) (FeCl_3_), and a thiol additive (2‐mercaptoethanol, βME, or dithiothreitol, DTT).^[14]^

**Figure 1:**
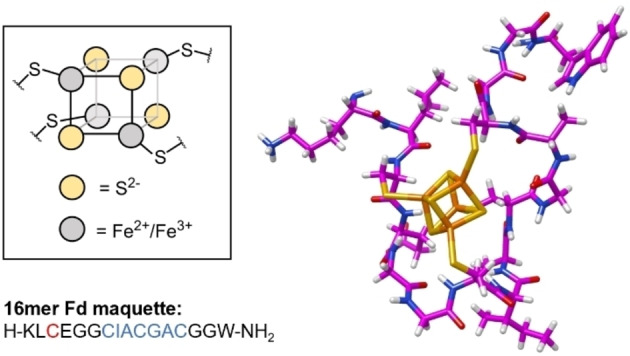
Schematic representation of the core cubic [4Fe4S] cluster and 3‐dimensional presentation of the [4Fe4S]‐peptide maquette for the 16mer sequence shown.

Efforts to explore and understand these systems have led to the preparation of short peptide maquettes that can be used as convenient models of the native protein.^[15]^ These synthetic analogues mimic the cluster‐forming region of the ferredoxins and enable the exploration of local steric and electronic variables on the formation and redox properties of the cluster. A 16mer maquette, derived from native ferredoxin sequences (Figure 1), has been demonstrated to yield effective cluster reconstitution under the same conditions as *apo* ferredoxin reconstitution.^[16]^ Interrogation of this synthetic system has enabled the fundamental protein ligand requirements of the cluster to be determined and explored.[^17^, ^18^] These studies demonstrate that the spacing of the Cys residues within the consensus sequence is immutable for cluster formation. However, variations of the non‐ligand residues yield cluster formation to varying degrees depending on the amino acid and location within the consensus sequence. Indeed, a [4Fe4S]‐cluster can be formed when the non‐ligand amino acids are all replaced with glycine (Gly).^[18]^

While non‐peptidic synthetic analogues of [4Fe4S] clusters have also been explored,^[19]^ peptide maquettes offer a fully synthetic system able to mimic the native environment of the FeS cluster, as well as the biological activity of the ferredoxins, in aqueous solution. Beyond the application of these maquettes to model proteins, only a handful of reports describe the potential of these artificial metalloproteins for synthetic biology and biotechnology.[^20^, ^21^] The ability to synthesize short peptides that can be produced at scale, and which reproducibly form redox‐active [4Fe4S] clusters, could be exploited to build synthetic and semi‐synthetic electron transfer systems.^[19]^

To explore such applications, we revisited several minimal peptide maquettes (Table 1) and further probed the reconstitution and ‘tuning’ of the redox potential of these clusters. Modulation of the cluster redox chemistry was attempted by switching a thiolate ligand carried by one Cys residue with the selenolate of selenocysteine (Sec). We demonstrate the application of these maquettes beyond the modeling of fundamental biology by substituting the physiological redox partner of a *Clostridium acetobutylicum* [FeFe]‐hydrogenase with a synthetic ferredoxin mimic to facilitate enzyme‐mediated H_2_‐oxidation.

**Table 1:** Peptide sequences, X‐band cw‐EPR, and CV data for maquettes **FdM1**, and **Mq2‐Mq6 (Sec)**.

Peptide	Sequence	*g*‐matrix^[b]^	Linewidths /mT^[c]^	H‐Strain /MHz^[c]^	% of reduced [4Fe4S]^+^ *wrt* **FdM1**	E_p,c_ (mV vs. SCE)^[d]^
**FdM1** ^[a]^	Ac‐KLCEGGCIACGACGGW‐NH_2_	[1.896 1.925 2.046]^1^ [1.868 1.935 2.071]^2^	[2.36 1.81] [2.07 1.0]	[43 0 0] [77 0 0]	100	−429
**Mq2**	Ac‐KLGEGGGIAGGAGGGW‐NH_2_	N/A	N/A	N/A	0	N/A
**Mq3**	Ac‐CGGGCGGCGGC‐NH_2_	[1.898 1.928 2.050]	[3.87 1.31]	[194 0 0]	21	ND
**Mq4**	Ac−GCGGGCGGCGGCG‐NH_2_	[1.898 1.931 2.050]	[3.92 0.92]	[180 20 130]	19	ND
**Mq5**	Ac−GCGGGCGGCGGCGY‐NH_2_	[1.887 1.927 2.056]	[2.62 2.15]	[196 3 130]	40	−471
**Mq6 (Sec)**	Ac−GUGGGCGGCGGCGY‐NH_2_	[1.877 1.912 2.075]	[4.44 1.87]	[156 0 150]	46	−476

[a] Simulation of the **FdM1** EPR spectrum involves inclusion of two EPR‐active species whose population is 1=66 % and 2=34 %; [b] For all the EPR simulations, the background subtraction leads to ambiguity in the position of the *“g*_3_/*g*_max_” feature, and gives an estimated error in the *“g*_3_/*g*_max_” value of ~±10 %; [c] The line shape of the EPR spectrum was reproduced by considering an isotropic Voigtian line shape and an anisotropic broadening (H‐Strain) respectively. The error in the estimated linewidths is ~±20 %. [d] Cyclic Voltammetry (CV) measured in V against SCE at room temp. in 50 mM HEPES, 10 mM KCl, 100 mM NaCl, pH 8.0 at room temperature. ND=not determined.

## Results and Discussion

**Figure 2:**
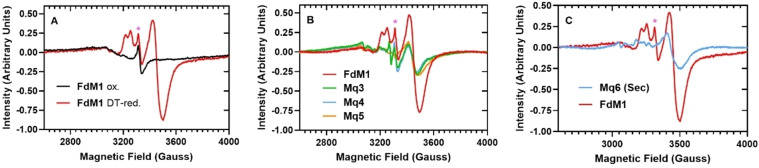
X‐band continuous‐wave (cw)‐EPR spectra (20 K) of the oxidized and one‐electron reduced maquettes. **A**. **FdM1** anaerobically reconstituted in the presence of βME, reduced with DT; **B**. **Mq3**, **Mq4**, **Mq5** anaerobically reconstituted in the absence of βME (and DT‐reduced), compared to DT‐reduced **FdM1**; **C**. **Mq6 (Sec)** anaerobically reconstituted in the presence of DTT (and DT‐reduced), compared to DT‐reduced **FdM1**. The sharp feature observed at 3350 G (highlighted with an asterisk) is attributed to a radical impurity present in the HEPES buffer.

As an initial model, a previously reported 16mer ferredoxin maquette was synthesized with an acetylated *N*‐terminus on Rink Amide resin (**FdM1**: Ac‐KLCEGGCIACGACGGW‐NH_2_, Table 1) via Fmoc‐SPPS (see SI for details). The **FdM1**‐[4Fe4S]^2+^ cluster was then reconstituted as previously described.^[16]^ Briefly, under anaerobic conditions, **FdM1** (1 μmol) was dissolved into 1760 μL of degassed HEPES buffer (50 mM HEPES, 10 mM KCl, pH 8.0) containing 2–10 vol % v/v 2‐mercaptoethanol (βME) at room temperature. FeCl_3_ (120 μL of a 50 mM solution) was added in 20×6 μL increments over a period of 30 mins. This was followed by addition of Na_2_S (120 μL of a 50 mM solution) in 10×12 μL increments over a period of 10 min (0.5 mM final peptide concentration). The solution was agitated for 60 mins, centrifuged, and the supernatant isolated. For control samples **FdM1**, was reconstituted under aerobic conditions, and under anaerobic conditions without addition of βME. Additionally, a non‐coordinating peptide based on **FdM1** with Gly residues replacing all the ligand Cys (**Mq2**) was also synthesized (Table 1 and SI) and subjected to the reconstitution protocol under anaerobic conditions. We found it challenging to achieve consistent cluster formation when eluting the samples through size exclusion columns (PD‐Midi Trap G10) to remove small molecule reagents, despite performing the elution in a glovebox and flushing the columns with degassed buffer prior to sample addition. Furthermore, our peptide maquettes are close to the size exclusion limit of these columns, and therefore complete separation from small molecule additives was not guaranteed. The ‘crude’ maquette samples were therefore frozen in liquid dinitrogen and analyzed by X‐band cw‐EPR at 20 K. Dithionite (DT)‐reduced **FdM1** reconstituted under anaerobic conditions gave the characteristic signals of a one‐electron reduced [4Fe4S]^+^ cluster (Figure 2A, Table 1, Entry 1, and Figures S7 and S8). The appearance of the doublet structure at the field ~3200–3300 G implies that there are two EPR active species present in the sample, plausibly due to different solvated populations of the reduced [4Fe4S]^+^ cluster. The spectrum was successfully simulated by considering two EPR contributing species and the extracted spin‐Hamiltonian parameters are given in Table 1. It is noteworthy that the extracted g‐matrix is in good agreement with previously reported values.[^17^, ^22^, ^23^] As expected, EPR of the anaerobically reconstituted oxidized maquettes (Figure 2A; black trace), the DT‐reduced aerobically reconstituted maquettes, and the anaerobically reconstituted non‐coordinating **Mq2** did not exhibit the EPR signals consistent with a reduced [4Fe4S]^+^ cluster. The **FdM1** control reconstituted under anaerobic conditions *without* the βME additive (and reduced with DT) did demonstrate some cluster formation (Figure S10). Comparison of the second integral of the two spectra gives 9 % cluster formation relative to the **FdM1** sample reconstituted with βME. Previous studies have interrogated the local steric and electrostatic environment of the ferredoxins around the conserved cluster‐forming region by preparing peptides with all amino acids other than Cys replaced with Gly.^[18]^ These maquette sequences have been shown to form clusters, albeit at a lower efficiency compared to the 16mer Fd motif. To explore these ‘minimal’ systems alongside **FdM1** we synthesized several poly‐glycine sequences,[^16^, ^18^] keeping the spacing of the Cys residues consistent with the conserved cluster‐forming region. **Mq3** has no residues flanking the first and fourth Cys positions, **Mq4** has Gly residues flanking these ‘terminal’ Cys, and **Mq5** has a tyrosine (Tyr) residue at the C‐terminal position as a UV‐absorbing handle to facilitate purification (Table 1). While reconstitution in the presence of βME clearly affords more efficient formation of the **FdM1** cluster, this additive must be removed via SEC under strictly anaerobic conditions prior to UV‐Vis characterization (to eliminate the ligand to metal charge transfer (LMCT) band of the thiol additive coordinating to Fe) and application of the cluster to any redox chemistry. As our **FdM1** control sample reconstituted in the absence of βME did show approximately 10 % cluster formation, and due to the stability/exclusion limit challenges we encountered when eluting the clusters through the size exclusion columns, we were motivated to explore the formation of maquettes **Mq3**–**Mq5** without this thiol additive. UV‐Vis analysis of these peptides reconstituted under anaerobic conditions without βME indicated successful formation by a strong Fe−S LMCT band at 420 nm (Figures S27–S30). EPR analysis of these samples (reduced with DT) confirmed [4Fe4S]^+^ cluster formation (Figure 2B and Figures S11–13). Despite the omission of βME, cluster incorporation was reasonable, and indeed higher than that of **FdM1** reconstituted without the thiol additive. Relative to the **FdM1** maquette reconstituted with βME, **Mq3**, **Mq4**, and **Mq5** clusters were formed in 21 %, 19 %, 40 % yield, respectively (Tables 1 and S1).

Cyclic voltammetry (CV) analysis of the **FdM1** and **Mq5** maquettes gave a single cathodic peak potential (E_p,c_) at more positive potentials than typically expected for these peptide maquettes.[^16^, ^24^] The E_p,c_ for **FdM1** was measured as −429 mV vs. SCE (−188 mV vs. SHE) and −471 mV vs. SCE (−230 mV vs. SHE) for **Mq5** (Figure S18–S20). Furthermore, the anodic current (i_p,a_) for both samples was significantly lower than the cathodic current (i_p,c_); the peak was also offset from the reduction process. Quasi‐reversible processes have previously been noted in synthetic [4Fe4S]^2+/+^ systems,^[20]^ however, the degree of irreversibility in our example is somewhat unusual. The shift of the E_p,a_ to a positive potential may indicate a perturbation of the structure of the cluster. Due to the non‐classical shape of the voltammogram, we cannot accurately determine mid‐point redox potentials (E_1/2_), but given the E_p,c_ values measured, the E_1/2_ for these maquettes are likely to sit at more positive potentials than those typically seen for the ferredoxin proteins (−300 to −700 mV vs. SHE^[7]^) and the previously reported non‐acetylated version of the **FdM1** maquette (−350 mV vs. SHE^[16]^). However, our data are closer to that of a similar 16mer ferredoxin‐like maquette, with a single Ala to Gly substitution relative to **FdM1** (−289 mV vs. SHE).^[25]^ Cyclic voltammetry was also carried out on control samples of peptide (**FdM1** and **Mq5**), Na_2_S, and FeCl_3_. In addition, the reagents for maquette formation were combined under *aerobic* conditions under which cluster formation was not expected (Figures S22–S26). Only the reagents combined under anaerobic conditions gave the CV plots discussed (Figures S18–S20). However, as these samples were not eluted through SEC, we cannot rule out alternative molecular species accounting for the lower than expected E_p,c_ and E_p,a_ values.

The difference in the potentials of **FdM1** and **Mq5** demonstrates that the local steric and electronic environment of the maquette modulates the redox properties of the cluster, as observed in previous studies.^[24]^ To further explore ‘tuning’ of the redox properties of the cluster, we synthesized **Mq6 (Sec)**; a similar sequence to **Mq5** with the first Cys residue switched to Sec. Known as the 21^st^ proteinogenic amino acid,^[26]^ Sec carries a selenol group as its sidechain with a redox potential significantly lower than that of the thiol of Cys (−381 mV for Sec compared to −180 mV for Cys[^26^, ^27^, ^28^]). Selenoproteins exist in all major domains of life,[^29^, ^30^] playing vital roles in redox signaling and as antioxidants. Sec as an Fe‐ligand in place of Cys occurs naturally in [NiFeSe]‐hydrogenases^[31]^ and has been explored via engineering of tRNA for selenocysteine insertion sequence (SECIS)‐independent Sec incorporation and synthetically, via peptide ligation.[^32^, ^33^] To explore the effect of direct Se‐incorporation into the [4Fe4S] cluster, a ferredoxin maquette,^[25]^ and select ferredoxin,^[34]^ hydrogenase,^[35]^ and nitrogenase proteins have been prepared hosting a [4Fe4Se] cluster.[^32^, ^36^] A Se substitution has also been demonstrated in a [2Fe2S]‐hydrogenase.^[37]^ To interrogate the effect of Sec on the redox properties of the [4Fe4S] cluster in peptide maquettes, [4Fe4S]‐**Mq6 (Sec)** was reconstituted anaerobically as described but with the addition of dithiothreitol (DTT) in place of βME to minimize the formation of seleno‐sulfides, which are likely interfere with cluster formation.^[38]^ Switching one of the Fe ligands to this alternative chalcogen had little effect on the redox potential of the cluster. The CV of this sequence gave an E_p,c_ of −476 mV vs. SCE (−235 mV vs. SHE; Figure S18 and S21), comparable to the value acquired for **Mq5**. The EPR spectrum of **Mq6 (Sec)** confirmed the presence of the [4Fe4S]^+^ cluster (Figure 2C), affording 46 % incorporation compared to **FdM1** reconstituted with βME (Table 1).

**Figure 3:**
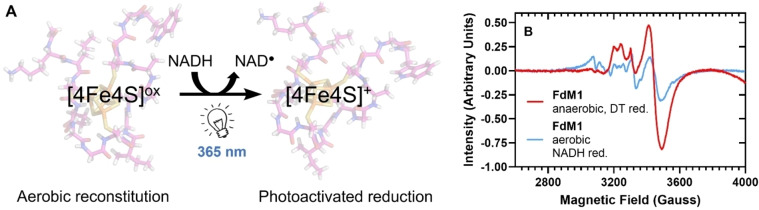
**A**. Reduction of aerobically reconstituted **FdM1** cluster using photoactivated NADH. **B**. X‐band frozen solution cw‐EPR spectra at 20 K of the one‐electron reduced, [4Fe4S]^+^ cluster reconstituted under anaerobic (red trace, reducing agent, DT=dithionite) and aerobic conditions (blue trace, reducing agent – photoactivated NADH).

Since the [4Fe4S] cluster is highly oxygen sensitive, ferredoxin proteins and peptide maquettes are reconstituted under strictly anaerobic conditions. As a control for our study, each maquette was also reconstituted aerobically. As expected, reduction of these aerobic controls with DT did not give the characteristic [4Fe4S]^+^ EPR signals, indicating that the cluster was not formed under ambient oxygen levels. Previous studies using photoactivated NADH as a more powerful reducing agent (relative to DT) for EPR studies of oxygenases^[39]^ prompted us to employ light‐activated electron transfer to interrogate the aerobically reconstituted samples further. We found that reconstitution of the **FdM1** and **Mq5** maquettes under aerobic conditions directly on the bench, with addition of NADH prior to freezing of the solution, followed by NADH activation under irradiation of the frozen solution at 365 nm, gave the characteristic EPR signal for the [4Fe4S]^+^ cluster (Figure 3B, **FdM1** sample). The %‐cluster incorporation was found to be 34 % relative to **FdM1** reconstituted under anaerobic conditions. Unsurprisingly, the peptide cluster was found to degrade quickly upon exposure to O_2_ resulting in formation of a precipitate and reduced absorbance at 420 nm. Oxidative degradation of FeS proteins has been well studied,[^19^, ^40^] thus cluster formation under ambient atmosphere is usually disregarded due to the O_2_‐sensitive nature of the clusters. The initial formation of the maquette clusters under aerobic conditions is, therefore, of interest.

Due to the low reduction potential of the [4Fe4S]^2+/+^ clusters, they fall within the redox window of hydrogen metabolism, and could potentially be used as components for H_2_‐technology in tandem with hydrogenase proteins. [FeFe]‐hydrogenases are redox metallo‐enzymes harbouring a specialized FeS cluster called the H‐cluster that reversibly catalyzes the oxidation of H_2_. The direction of this redox process depends on a redox coupling partner, which the enzyme requires to complete the cycle.^[41]^ In the case of the [FeFe]‐hydrogenase CaHydA from *Clostridium acetobutylicum*, the natural redox partner is a bacterial‐type 2[4Fe4S] ferredoxin that is expected to bind to the *N*‐terminus of the enzyme via the so‐called F‐domain. The turnover frequency (TOF; kcat) for this physiologic process has been calculated to be 901±225 s^−1^.[^42^, ^43^] We sought to substitute the natural coupling partner for this H_2_‐oxidation process with a [4Fe4S]‐peptide maquette. In this system, the CaHydA‐mediated oxidation of H_2_ can be monitored by UV‐Vis spectroscopy via reduction in the [4Fe4S]‐cluster's characteristic LMCT absorbance at 420 nm.

To explore this semi‐synthetic redox coupling, we reconstituted the **FdM1** maquette under the described anaerobic conditions in the presence of βME. The reconstituted **FdM1** cluster was eluted through a size exclusion column (PD MidiTrap G10) under anaerobic conditions, and the sample was transferred into a quartz cuvette and sealed. Despite our previous challenges regarding SEC elution, UV‐Vis analysis of the sample gave the characteristic cluster absorbance at 420 nm. The solution was sparged with hydrogen for 5 minutes. Analysis of the sample by UV‐Vis showed a stable absorbance at 420 nm. A stock of *Clostridium acetobutylicum* hydrogenase (CaHydA)[^41^, ^44^] was prepared in a sealed vial (0.2 mg/mL); an aliquot of the enzyme was transferred to the H_2_‐maquette solution via gastight syringe (final concentration of the enzyme 0.5 μg/mL, final concentration of **FdM1**, 0.25 mM), and the sample re‐analyzed via UV‐Vis. A decrease in absorbance at 420 nm, measured over a 10‐minute period, demonstrated successful coupling of our maquette into this semi‐synthetic H_2_‐oxidation pathway as the terminal electron acceptor (Figure S32). Once the absorbance of the electron transfer had plateaued, the system was exposed to O_2_. As expected, subsequent addition of DT gave no further reduction in absorbance (Figure S33).

**Figure 4:**
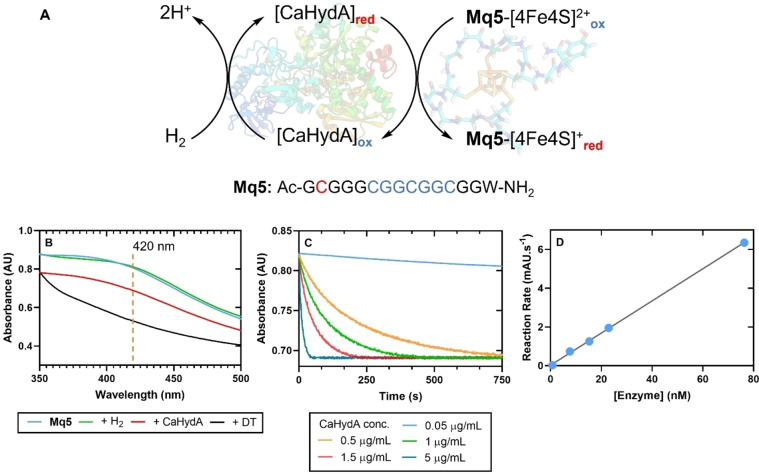
**A**. Integration of **Mq5** as the terminal electron acceptor within a hydrogenase catalyzed H_2_‐oxidation pathway. **B**. UV‐Vis analysis of **Mq5**, followed by sequential addition of H_2_, CaHydA, and DT (dithionite); Abs.=420 nm; **C**. Rate plot comparing reactions run with increasing concentration of *Clostridium acetobutylicum* hydrogenase (CaHydA); **Mq5** peptide concentration 0.25 mM; CaHydA conc.: 0.05 μg/mL=0.76 nM; 0.5 μg/mL=7.6 nM; 1 μg/mL=15.3 nM; 1.5 μg/mL=22.9 nM; 5.0 μg/mL=76.3 nM. **D**. Plot of the enzyme conc. versus initial reaction gradient (R^2^=0.998).

We then sought to repeat this result with the minimal peptide maquette, **Mq5**. Since we have demonstrated successful cluster formation in the absence of βME to a satisfactory level of reconstitution for this maquette (40 % *wrt* the **FdM1** reconstituted with βME), the hydrogenase‐mediated oxidation of H_2_ was repeated using this peptide reconstituted in the absence of βME eliminating the requirement to remove the thiol additive via SEC. UV‐Vis analysis of the reconstituted maquette gave the characteristic LMCT absorbance at 420 nm (Figure S30). As before, sparging of the solution with H_2_ did not change the UV‐Vis profile (Figure 4B). Upon addition of the enzyme (to a final concentration of 0.5 μg/mL) to the H_2_‐sparged **Mq5** solution (concentration 0.25 mM *wrt* **Mq5**), the UV absorbance at 420 nm again decreased over 10 mins to a steady value (Figure 4C).

Further addition of enzyme and re‐sparging with H_2_ did not lead to a further decrease in absorbance. Without first exposing the reaction to ambient O_2_, addition of the reducing agent (DT) decreased the absorbance further, indicating that the **Mq5** cluster was not completely reduced by the CaHydA in this process (Figure 4B). The experiment was repeated at various concentrations of CaHydA (Figure 4C: 0.05, 1, 1.5, and 5 μg/mL). In all repeats but 0.05 μg/mL (which gave a very shallow decrease in absorbance), the absorbance at 420 nm decreased rapidly with addition of the enzyme. A plot of the initial slope of the UV‐Vis decrease shows a linear dependence on the concentration of enzyme, confirming that the synthetic maquette can directly interact with the enzyme resulting in productive electron transfer (Figure 4D). The TOF for our electron transfer process was calculated as 79±5.8 s^−1^, assuming cluster concentration equal to that of the peptide concentration of the sample. However, based on the comparison of EPR data for both the **FdM1** cluster reconstituted with βME and **Mq5** cluster reconstituted without this thiol additive, the extent of **Mq5** cluster formation appears to be approximately 40 % relative to **FdM1**, which would afford a TOF value of 31±2.3 s^−1^. We can therefore conclude that the TOF of our semi‐synthetic system is *at least* one order of magnitude lower than the physiological redox partnership, which exploits two [4Fe4S] clusters.[^42^, ^43^]

As described, each experiment plateaued at the same absorbance (Figure 4B); while these data could indicate that a redox equilibrium has been established between the H_2_/H^+^ redox couple and the maquette, the CV analysis of the maquette clusters does not support this conclusion. However, the fact that the cluster is not fully reduced does indicate that the redox potential for these clusters is closer to, or more negative than, that of the redox midpoint for hydrogen, as previously reported,[^16^, ^24^] which would enable an equilibrium to be established. Thus further interrogation of these synthetic redox mediators is required to fully understand the electrochemistry involved.

## Conclusions

We have demonstrated the integration of a fully synthetic ferredoxin‐inspired peptide‐[4Fe4S]^2+/+^ cluster as the redox partner for an [FeFe]‐hydrogenase. The maquette is employed to complete the redox cycle of hydrogenase‐mediated H_2_‐oxidation, coupling our synthetic cluster with the FeS clusters of the hydrogenase protein. Due to the ease of preparation of these peptide clusters, and the tolerance to deviation of the non‐ligand residues, such synthetic motifs could be used as versatile tools to shuttle electrons within artificial/semi‐synthetic biological pathways. Further interrogation of these systems may lead to interesting applications within synthetic biology.

## Experimental Section

**General**: High‐resolution mass spectra were recorded on a Bruker MicroTOF Focus II MS (ESI) operating in positive or negative ionisation mode. Analytical HPLC was performed on a Thermo Ultimate 3000 mHPLC system equipped with PDA eλ detector (λ=210–400 nm). Peptides were analyzed using a Waters Sunfire 5 μm, 2.1×150 mm column (C‐18) at a flow rate of 0.6 mL/min. The mobile phase was composed of 0.1 % trifluoroacetic acid in H_2_O (Solvent A) and 0.1 % trifluoroacetic acid in acetonitrile (Solvent B). The analysis of the chromatograms was conducted using Chromeleon 7 software (gradients specified in the SI). Preparative reverse‐phase HPLC was performed using a Waters 1525 binary pump HPLC equipped with a dual wavelength UV detector set to 210 nm and 280 nm. Peptides were purified using either a Waters Sunfire 5 μm, 10×250 mm (C‐18) semi‐preparative column, operating at a flow rate of 5 mL/min, or a Waters Sunfire 5 μm, 19×150 mm (C‐18) preparative column, operating at a flow rate of 6 mL/min using a mobile phase of 0.1 % trifluoroacetic acid in water (Solvent A) and 0.1 % trifluoroacetic acid in acetonitrile (Solvent B) (gradients specified in the SI). All buffers were made up using MilliQ water and degassed using the freeze‐pump‐thaw method.

**Materials** Commercial materials were used as received unless otherwise noted. Amino acids, coupling reagents, and resins were obtained from Novabiochem, Fluorochem or GL Biochem. Amino acids were purchased as Fmoc‐protected (l)‐amino acids with appropriate, acid‐labile sidechain protecting groups. Reagents that were not commercially available were synthesized as outlined in the SI. Solvents were obtained as reagent grade from Merck or Fisher. See the SI for details regarding compound synthesis.

### Solid Phase Peptide Synthesis (SPPS)

#### Manual Fmoc‐SPPS

*Preloading Rink Amide resin*: Rink amide resin was initially washed with DCM (5×3 mL) followed by removal of the Fmoc group by treatment with 20 % piperidine/DMF (2×5 min). The resin was washed with DMF (5×3 mL), DCM (5×3 mL) and DMF (5×3 mL). Oxyma Pure (4 eq.) and DIC (4 eq.) were added to a solution of Fmoc‐AA‐OH carrying appropriate acid‐labile sidechain protection (4 eq.) in DMF. After 5 min of pre‐activation, the mixture was added to the resin. After 2 h the resin was washed with DMF (5×3 mL), DCM (5×3 mL) and DMF (5×3 mL), capped with acetic anhydride/pyridine (1 : 9 v/v) (2×3 min) and washed with DMF (5×3 mL), DCM (5×3 mL) and DMF (5×3 mL).

*Estimation of amino acid loading*: The resin was treated with 20 % piperidine/DMF (2×3 mL, 3 min) and 20 μL of the combined deprotection solution was diluted to 10 mL using 20 % piperidine/DMF in a volumetric flask. The UV absorbance of the resulting piperidine‐fulvene adduct was measured (λ=301 nm, ϵ=7800 M^−1^ cm^−1^) to determine loading of the resin.

*General amino acid coupling*: A solution of Fmoc‐AA‐OH carrying appropriate acid‐labile sidechain protection (4 eq.), DIC (4 eq.) and Oxyma Pure (4 eq.) in DMF (final concentration 0.1 M) was added to the resin. After 1 h, the resin was washed with DMF (5×3 mL), DCM (5×3 mL) and DMF (5×3 mL).

*Capping*: Acetic anhydride/pyridine (1 : 9 v/v) was added to the resin (3 mL). After 3 min the resin was washed with DMF (5×3 mL), DCM (5×3 mL) and DMF (5×3 mL).

*Fmoc deprotection*: The resin was treated with 20 % piperidine/DMF (2×3 mL, 3 min) and washed with DMF (5×3 mL), DCM (5×3 mL) and DMF (5×3 mL).

*Cleavage*: A mixture of TFA, thioanisole, tri*iso*propylsilane (TIS) and water (90 : 4 : 4 : 2 v/v/v/v) was added to the resin. After 3 h, the resin was washed with TFA (3×2 mL). For peptide containing Sec (U): a mixture of trifluoroacetic acid (TFA), trimethylsilyl trifluoromethanesulfonate (TMSOTf), thioanisole, and *m*‐cresol (66 : 18 : 11 : 5 v/v/v/v) was added to the dried resin and shaken at −4 °C for 1 h. The resin was washed with TFA (3×2 mL).

*Work‐up*: The combined cleavage solutions were concentrated under a stream of dinitrogen to <5 mL. 40 mL of diethyl ether was added to precipitate the peptide and the suspension centrifuged. The pellet was then dissolved in water containing 0.1 % TFA, filtered and purified by preparative HPLC and analyzed by – analytical HPLC and ESI mass spectrometry.

**Automated Solid‐Phase Peptide Synthesis** Automated Fmoc‐SPPS was carried out on either a Biotage Initiator^+^ Alstra or CEM Liberty Blue microwave peptide synthesizer. General synthetic procedures for Fmoc‐deprotection and capping were carried out in accordance with the manufacturer's specifications. Biotage Initiator^+^ Alstra: standardized amino acid couplings were performed for 15 min at 50 °C under microwave irradiation in the presence of amino acid (0.5 M in DMF, 4 eq.), Oxyma Pure (0.5 M in DMF, 4 eq.) and di*iso*propylcarbodiimide (0.5 M in DMF, 4 eq.). Peptide cleavage and work‐up were carried out as described above for manual SPPS. CEM Liberty Blue: standardized amino acid couplings were performed for 2.5 min at 90 °C under microwave irradiation in the presence of amino acid (0.2 M in DMF, 4 eq.), Oxyma Pure (1 M in DMF, 4 eq.) and di*iso*propylcarbodiimide (1 M in DMF, 4 eq.). Peptide cleavage and work‐up were carried out as described above for manual SPPS.

**Reconstitution of cluster maquettes** All steps of the incorporation of iron‐sulfur clusters into the required apo‐peptide were performed anaerobically in an MBraun UNIlab LMF Glovebox Workstation equipped with a Gas Purifier MB20/MB200 G at O_2_ concentrations≤0.1 ppm. All solutions and buffers were degassed and purged with argon prior to use. Methodologies for the assembly protocol were adapted from previous studies.^[17]^ Stock solutions of FeCl_3_ and Na_2_S were freshly prepared in anaerobic conditions in 50 mM HEPES, 10 mM KCl, pH 8.0 buffer at concentration of 50 mM. Peptide (1 μmol) was equilibrated under gentle stirring in HEPES buffer (50 mM HEPES, 10 mM KCl, pH 8.0) containing 2–10 vol % (v/v) of 2‐mercaptoethanol (final volume 1760 μL) at r.t. for 5 min. FeCl_3_ (120 μL of a 50 mM stock solution) was added in twenty increments (20×6 μL) over a period of 30 min. Na_2_S (120 μL of a 50 mM stock solution) was added in ten increments (10×12 μL) over a period of 10 min (0.5 mM final peptide concentration, 3 mM final FeCl_3_ and Na_2_S concentrations). The reaction mixture was equilibrated under gentle stirring at r.t. for 1 hour, before being centrifuged (13,000 rpm for 5 min). The supernatant was discharged and the solution was transferred into an Eppendorf tube. When required, reconstituted samples were purified using PD MidiTrap G‐10 size exclusion columns under strict anaerobic conditions.

For samples prepared without βME, HEPES buffer without the thiol additive was prepared. For reconstitution of **Mq6 (Sec)**, βME was replaced in the HEPES buffer by 5 equiv. of DTT *wrt* peptide.

**UV‐Vis spectroscopic analysis of [Fe‐S] cluster reconstitutions**: The instrument used was a Cary 5000 UV‐Vis‐NIR. All measurements were performed in quartz cuvettes with 1 cm optical path length. Both blank and sample solutions were made up freshly in the glove box following previous guidelines and then immediately analysed. The range of wavelength investigated was from 300 nm to 600 nm. Samples were diluted in 50 mM HEPES, 10 mM KCl, pH 8.0 buffer to a final concentration of 90 μM and then analysed.

**Electron Paramagnetic‐Resonance spectroscopy (EPR)**: All samples were measured on a Bruker EMXplus EPR spectrometer equipped with a Bruker ER 4112SHQ X‐band resonator. Sample cooling was achieved using a Bruker Stinger^[45]^ cryogen free system mated to an Oxford Instruments ESR900 cryostat; temperature control was maintained using an Oxford Instruments MercuryITC. The optimum conditions used for recording the spectra are given below; microwave power 20 dB (2.19 mW), modulation amplitude 5 G, time constant 82 ms, conversion time 30 ms, sweep time 90 s, receiver gain 30 dB and an average microwave frequency of 9.385 GHz. The photo‐reduction of **FdM1** was performed in the presence of NADH (nicotinamide adenine dinucleotide) between ~230–240 K by placing the quartz EPR tube containing the sample in a 1‐propanol and dry‐ice/liquid dinitrogen solvent mixture. Optical irradiation at 365 nm was accomplished for 30 minutes using a Thorlabs Mounted High Power LED (M365 L3) with the output beam collimated using a Thorlabs collimation adaptor (SM2F32‐A). Optimal output (1.3 W typical) was maintained by driving with a constant current of 1 A from a Thorlabs LED Driver (LEDD1B). All samples were measured as a frozen solution at 20 K. The analysis of the continuous wave EPR spectra was performed using the EasySpin toolbox^[46]^ for the Matlab program package. EPR samples were prepared, unless otherwise indicated, in strict anaerobic conditions in an MBraun UNIlab LMF Glovebox Workstation at O_2_ concentrations≤0.1 ppm. All samples contained 10 % glycerol as cryoprotectant. Aliquots of freshly reconstituted maquettes (final concentration 250 μM based on peptide) were reduced using a freshly prepared solution of sodium dithionite (500 μM final DT concentration). After 5 minutes of incubation with sodium dithionite, samples were transferred into EPR tubes, capped with rubber septa, and flash frozen in liquid N_2_. EPR samples were stored in liquid N_2_ prior to data collection.

**Cyclic voltammetry**: Experiments were performed under strictly anaerobic conditions at room temperature on a CH Model 600E Series Potentiostat/Galvanostat with a three‐electrode cyclic voltammetry configuration. A saturated calomel electrode (SCE) was used as the reference electrode. A platinum wire was used as the counter electrode. A glassy carbon electrode was used as the working electrode. Prior to experiments, the working electrode was polished with alumina slurry starting with 1 μm, followed by 0.3 μm, and 0.05 μm particles. 100 mM NaCl was added to all samples (maquettes initially reconstituted in 50 mM HEPES, 10 mM KCl, pH 8.0) as a supporting electrolyte. A CHI600E Electrochemical Analyzer was used for data manipulation. Redox experiments of the [4Fe‐4S]‐cluster maquette were evaluated by monitoring faradaic current for both oxidation and reduction during ten cycles across the range of −1.2 and +0.6 V (scan rate 100 mV/s, working electrode area: 0.071 cm^2^).

**General protocol for [4Fe‐4S]‐maquette reduction via [FeFe]‐hydrogenase‐mediated H_2_‐oxidation** Samples were handled in a Don Whitley A85 TG Anaerobic Workstation and analysed on a Shimadzu UV‐2600 Spectrophotometer. The [FeFe]‐hydrogenase used in these experiments was CaHydA from *Clostridium acetobutylicum*, obtained by recombinant overexpression and purification as previously described.[^41^, ^44^] After reconstitution, [4Fe‐4S]‐cluster maquettes were diluted x4, transferred into a quartz cuvette and sealed with a rubber turnover stopper. Samples were saturated with hydrogen by bubbling for 5 minutes. The reaction was started by adding an aliquot of CaHydA using a gastight syringe (Hamilton) from a 0.2 mg/mL stock to a desired enzyme concentration (in μg/mL). The final conc. of the peptide was 0.25 mM for all experiments. The reaction was monitored by UV‐vis spectroscopy at room temperature.

## Supporting Information

Additional references cited within the Supporting Information.[^17^, ^23^, ^41^, ^44^, ^45^, ^46^, ^47^]

## Conflict of interest

The authors declare no conflict of interest.

1

## Biographical Information

*Andrea Bombana was born in 1994 in Milan, Italy. In 2017 he completed his undergraduate studies in Chemistry and Industrial Chemistry at the University of Insubria in Como, Italy. In 2019 he received an MSc in Pharmaceutical Science and Medicinal Chemistry from Loughborough University, where he worked in the laboratory of Dr George W. Weaver. Andrea then moved to the University of Nottingham to undertake his PhD under the supervision of Dr Nicholas Mitchell. In 2022 he joined the CRO Domainex, where he currently works as a Senior Scientist I in the medicinal chemistry division*.

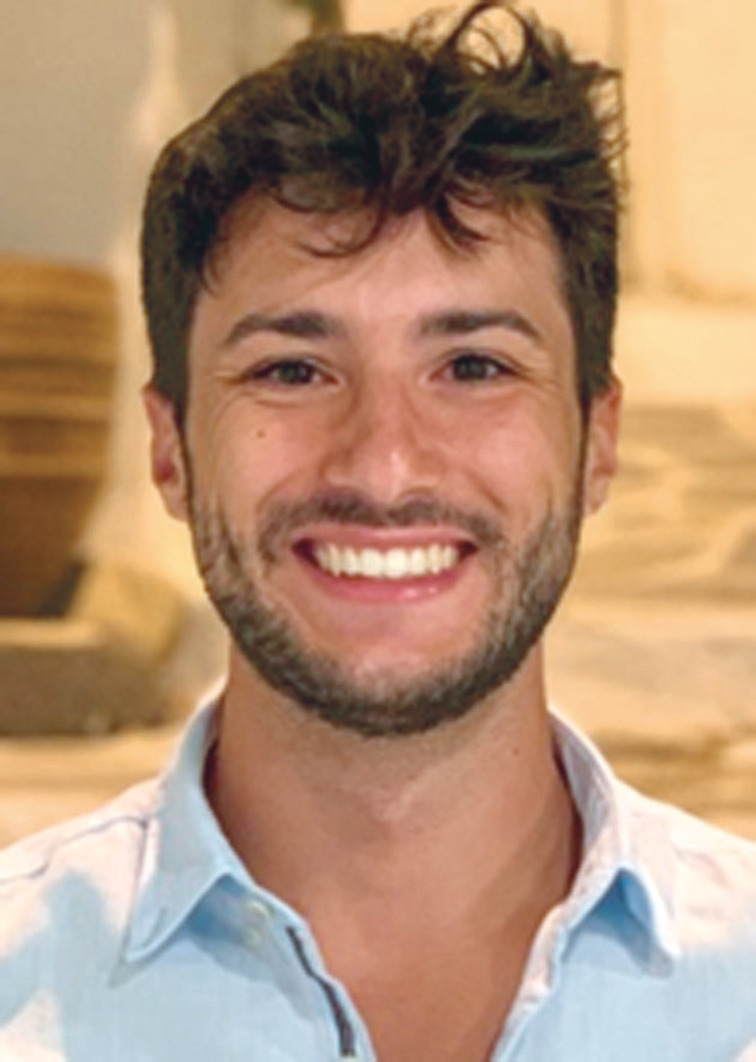

## Biographical Information

*Nick Mitchell received his PhD in 2010 from University College London (UCL), under the supervision of Prof. Stefan Howorka. He remained at UCL to work with Prof. Alethea Tabor and Prof. Helen Hailes before moving to the University of Sydney to work with Prof. Richard Payne. He returned to the UK in 2016 as a Nottingham Research Fellow to start his independent career and was promoted to Assistant Professor in 2019. Dr Mitchell's research interests include the development of chemistry to enable the site‐selective modification of peptides and proteins, and the exploration of peptide therapeutics and catalysts inspired by nature*.

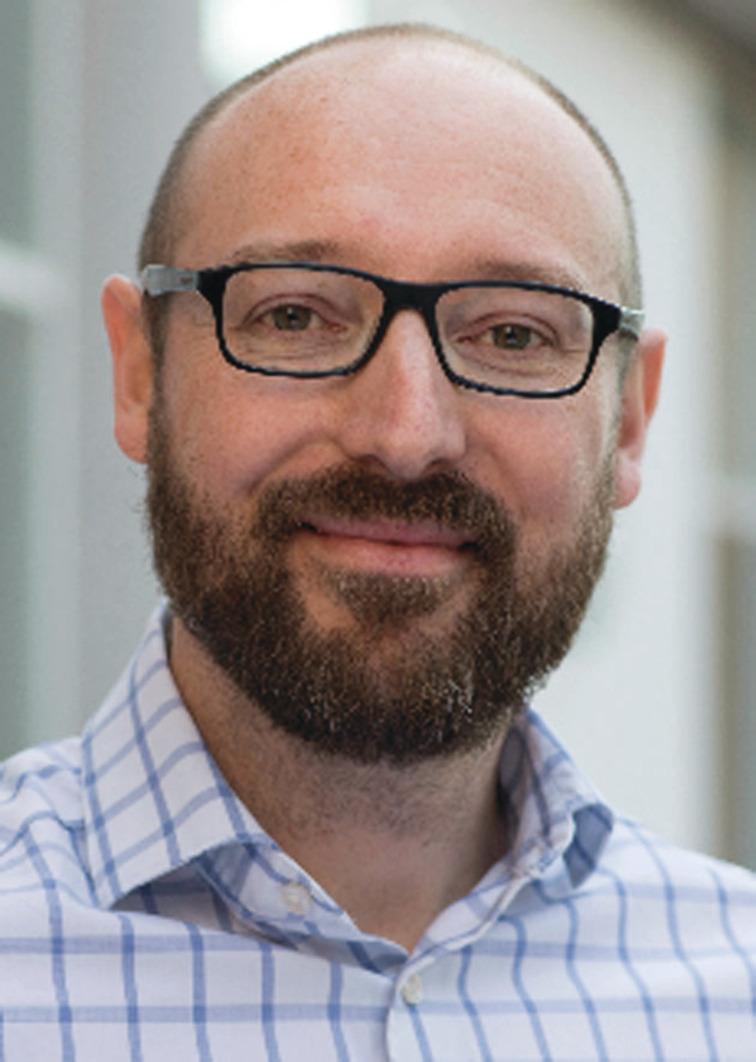

## Supporting information

As a service to our authors and readers, this journal provides supporting information supplied by the authors. Such materials are peer reviewed and may be re‐organized for online delivery, but are not copy‐edited or typeset. Technical support issues arising from supporting information (other than missing files) should be addressed to the authors.

Supporting Information

## Data Availability

The data that support the findings of this study are available in the supplementary material of this article.
