# Supplementary material for: Application of a Synthetic Ferredoxin‐Inspired [4Fe4S]‐Peptide Maquette as the Redox Partner for an [FeFe]‐Hydrogenase
Source: Chembiochem. 2023 Aug 13;24(18):e202300250. doi: 10.1002/cbic.202300250 (PMC10946529; doi:10.1002/cbic.202300250)
Supplement: Supplementary file 1 — Supporting Information [file CBIC-24-0-s001.pdf]

ChemBioChem

Supporting Information

Application of a Synthetic Ferredoxin-Inspired [4Fe4S]-Peptide Maquette as the Redox Partner for an [FeFe]-Hydrogenase

Andrea Bombana, Muralidharan Shanmugam, David Collison, Alexander J. Kibler, Graham N. Newton, Christof M. Jäger, Anna K. Croft, Simone Morra, and Nicholas J. Mitchell*

Contents

General Methods 2

Materials 2

Solid Phase Peptide Synthesis (SPPS) 3

Manual Fmoc-SPPS 3

Automated Solid-Phase Peptide Synthesis 4

Electrochemical Measurements 4

UV-Vis Spectroscopy 5

Electron Paramagnetic Resonance (EPR) Spectroscopy 5

General Reconstitution Protocol for [4Fe-4S] Maquettes 6

General Protocol for H_2-oxidation Experiments 6

Peptide Synthesis 8

EPR data 14

FdM1-[4Fe4S] 14

Mq3-[Fe_4S_4] 17

Mq4-[Fe_4S_4] 18

Mq5-[Fe_4S_4] 18

Mq6 (Sec)-[Fe_4S_4] 19

Estimation of $[4Fe\text{-}4S]^+$ Formation Relative to FdM1 20

Cyclic Voltammetry of Peptide-$[4Fe4S]^{2+/+}$ Clusters 25

UV-Vis Analysis of Peptide-$[4Fe4S]^{2+/+}$ Clusters 30

References 34

General Methods

High-resolution mass spectra were recorded on a Bruker MicroTOF Focus II MS (ESI) operating in positive or negative ionisation mode. Analytical HPLC was performed on a Thermo Ultimate 3000 uHPLC system equipped with PDA eλ detector (λ = 210 – 400 nm). Peptides were analyzed using a Waters Sunfire 5 μm, 2.1 × 150 mm column (C-18) at a flow rate of 0.6 mL•min^{-1}. The mobile phase composed of 0.1% trifluoroacetic acid in H_2O (Solvent A) and 0.1% trifluoroacetic acid in acetonitrile (Solvent B). The analysis of the chromatograms was conducted using Chromeleon 7 software.

Preparative reverse-phase HPLC was performed using a Waters 1525 binary pump HPLC equipped with a dual wavelength UV detector set to 210 nm and 280 nm. Peptides were purified on a Waters Sunfire 5 μm (C-18) preparative column with 5-μm particle size, 19 × 150 mm, operating at a flow rate of 6 mL•min^{-1} using a mobile phase of 0.1% trifluoroacetic acid in water (Solvent A) and 0.1% trifluoroacetic acid in acetonitrile (Solvent B) using the gradient specified in the experimental section. Semi-preparative reverse-phase HPLC was performed using the same HPLC and solvent system. The column used was a Waters Sunfire 5 μm (C-18) preparative column, 10 × 250 mm, operating at a flow rate of 5 mL•min^{-1} using the gradient specified in the experimental section.

Materials

Commercial materials were used as received unless otherwise noted. Amino acids, coupling reagents and resins were obtained from Novabiochem, Fluorochem or GL Biochem. Reagents that were not commercially available were synthesized as outlined in the experimental section. Solvents were obtained as reagent grade from Merck or Fisher. All aqueous solutions used for cluster formation were degassed via the freeze-pump-thaw method.

Solid Phase Peptide Synthesis (SPPS)

Manual Fmoc-SPPS

Preloading Rink Amide resin: Rink amide resin was initially washed with DCM (5 × 3 mL) followed by removal of the Fmoc group by treatment with 20% piperidine/DMF (2 × 5 min). The resin was washed with DMF (5 × 3 mL), DCM (5 × 3 mL) and DMF (5 × 3 mL). Oxyma Pure (4 eq.) and DIC (4 eq.) were added to a solution of Fmoc-AA-OH (carrying appropriate acid-labile sidechain protection) (4 eq.) in DMF. After 5 min of pre-activation, the mixture was added to the resin. After 2 h the resin was washed with DMF (5 × 3 mL), DCM (5 × 3 mL) and DMF (5 × 3 mL), capped with acetic anhydride/pyridine (1:9 v/v) (2 × 3 min) and washed with DMF (5 × 3 mL), DCM (5 × 3 mL) and DMF (5 × 3 mL).

Estimation of amino acid loading: The resin was treated with 20% piperidine/DMF (2 x 3 mL, 3 min) and 20 µL of the combined deprotection solution was diluted to 10 mL using 20% piperidine/DMF in a volumetric flask. The UV absorbance of the resulting piperidine-fulvene adduct was measured (λ = 301 nm, ε = 7800 M^{-1} cm^{-1}) to determine the amount of amino acid loaded onto the resin.

General amino acid coupling: A solution of Fmoc-AA-OH (carrying appropriate acid-labile sidechain protection) (4 eq.), DIC (4 eq.) and Oxyma Pure (4 eq.) in DMF (final concentration 0.1 M) was added to the resin. After 1 h, the resin was washed with DMF (5 × 3 mL), DCM (5 × 3 mL) and DMF (5 × 3 mL).

Capping: Acetic anhydride/pyridine (1:9 v/v) was added to the resin (3 mL). After 3 min the resin was washed with DMF (5 × 3 mL), DCM (5 × 3 mL) and DMF (5 × 3 mL).

Deprotection: The resin was treated with 20% piperidine/DMF (2 x 3 mL, 3 min) and washed with DMF (5 × 3 mL), DCM (5 × 3 mL) and DMF (5 × 3 mL).

Cleavage: A mixture of TFA, thioanisole, tri*iso*propylsilane (TIS) and water (90:4:4:2 v/v/v/v) was added to the resin. After 3 h, the resin was washed with TFA (3 × 2 mL). For peptide containing Sec (U), a mixture of trifluoroacetic acid (TFA), trimethylsilyl trifluoromethanesulfonate (TMSOTf), thioanisole, and *m*-

cresol (66:18:11:5 v/v/v/v) was added to the dried resin and shaken at - 4°C for 1 h. The resin was washed with TFA (3 x 2 mL).

Work-up: The combined solutions were concentrated under a stream of nitrogen to < 5 mL. 40 mL of diethyl ether was added to precipitate the peptide and the suspension centrifuged. The pellet was then dissolved in water containing 0.1% TFA, filtered and purified by preparative HPLC and analyzed by LC-MS and ESI mass spectrometry.

Automated Solid-Phase Peptide Synthesis

Automated Fmoc-SPPS was carried out on either a Biotage Initiator$^+$ Alstra or CEM Liberty Blue microwave peptide synthesizer. General synthetic procedures for Fmoc-deprotection and capping were carried out in accordance with the manufacturer's specifications. Biotage Initiator$^+$ Alstra: standardized amino acid couplings were performed for 15 min at 50 ºC under microwave irradiation in the presence of appropriately protected amino acid (0.5 M in DMF, 4 eq.), Oxyma Pure (0.5 M in DMF, 4 eq.) and di*iso*propylcarbodiimide (0.5 M in DMF, 4 eq.). Peptide cleavage and work-up were carried out as described above for manual SPPS. CEM Liberty Blue: standardized amino acid couplings were performed for 2.5 min at 90 ºC under microwave irradiation in the presence of amino acid (0.2 M in DMF, 4 eq.), Oxyma (1 M in DMF, 4 eq.) and di*iso*propylcarbodiimide (1 M in DMF, 4 eq.). Peptide cleavage and work-up were carried out as described above for manual SPPS.

Electrochemical Measurements

Experiments were performed under strictly anaerobic conditions at room temperature on a CH Model 600E Series Potentiostat/Galvanostat with a three-electrode cyclic voltammetry configuration. Saturated calomel electrode (SCE) was used as the reference electrode. A platinum wire was used as the counter electrode. Glassy carbon electrode was used as the working electrode. Prior to experiments, the working electrode was polished with alumina slurry starting with 1 µm, followed by 0.3 µm, and 0.05 µm particles. 100 mM NaCl was added to all samples as a supporting electrolyte. CHI600E Electrochemical Workstation was used for data acquisition. CHI600E Electrochemical Analyzer

was used for data manipulation. Redox experiments of the [4Fe-4S]-cluster maquette were evaluated by monitoring faradaic current for both oxidation and reduction during ten cycles across the range of -1.2 and +0.6 V (scan rate 100 mV/s; working electrode area: 0.071 cm^2).

UV-Vis Spectroscopy

Experiments were performed at room temperature on a Cary 5000 UV-Vis-NIR spectrophotometer. All measurements were performed in gas-tight quartz cuvettes with 1 cm optical path length. All blank and sample solutions were freshly prepared in anaerobic conditions and immediately analysed.

Electron Paramagnetic Resonance (EPR) Spectroscopy

EPR samples were prepared, unless otherwise indicated, in strict anaerobic conditions in an MBraun UNIlab LMF Glovebox Workstation at O_2 concentrations ≤ 0.1 ppm. All samples contained 10% glycerol as cryoprotectant. Aliquots of freshly reconstituted maquettes (final concentration 250 µM based on peptide) were reduced using a freshly prepared solution of sodium dithionite (final concentration 500 µM). After 5 minutes of incubation with sodium dithionite, samples were transferred into 4 mm outer diameter/3 mm inner diameter Suprasil quartz EPR tubes (Wilmad LabGlass) capped with rubber septa, and flash frozen in liquid N_2. The photo-reduction of **FdM1** was performed in the presence of NADH (nicotinamide adenine dinucleotide) between ~ 230-240 K by placing the quartz EPR tube containing the sample in a 1-propanol and dry-ice/liquid nitrogen solvent mixture. Optical irradiation at 365 nm was accomplished for an hour using a Thorlabs Mounted High Power LED (M365L3) with the output beam collimated using a Thorlabs collimation adaptor (SM2F32-A). Optimal output (1.3 W typical) was maintained by driving with a constant current of 1A from a Thorlabs LED Driver.

All EPR samples were measured on a Bruker EMXplus EPR spectrometer equipped with a Bruker ER 4112SHQ X-band resonators. Sample cooling was achieved using a Bruker Stinger[45] cryogen free system mated to an Oxford Instruments ESR900 cryostat, temperature control was maintained using an Oxford Instruments MercuryITC. The optimum conditions used for recording the

spectra are as follows: microwave power 20 dB (2.19 mW), modulation amplitude 5 G, time constant 82 ms, conversion time 30 ms, sweep time 90 s, receiver gain 30 dB and an average microwave frequency of 9.385 GHz. All EPR spectra were measured as a frozen solution at 20 K. The analysis of the continuous wave EPR spectra were performed using EasySpin toolbox (5.2.35) for the Matlab program package.[46]

General Reconstitution Protocol for [4Fe-4S] Maquettes

Methodologies for cluster assembly into the required peptide were adapted from previous protocols.[17] All steps of the reconstitution procedure were performed, unless otherwise indicated, in strict anaerobic conditions in an MBraun UNIlab LMF Glovebox Workstation at O_2 concentrations ≤ 0.1 ppm. All solutions and buffers were degassed via the freeze-pump-thaw method. Peptide (1 µmol) was equilibrated under gentle stirring with 2-mercaptoethanol (2-10% v/v of final solution) in HEPES buffer (final volume 1760 µL) at rt for 30 min. $FeCl_3$ (120 µL of a 50 mM solution) was added in twenty increments (20 x 6 µL) over a period of 30 min. Na_2S (120 µL of a 50 mM solution) was added in ten increments (10 x 12 µL) over a period of 10 min. The reaction mixture was equilibrated under gentle stirring at rt for 1 hour, before being centrifuged (13,000 rpm for 5 min). The supernatant was discharged, and the solution was transferred into an Eppendorf tube. When required, reconstituted samples were purified using PD MidiTrap G-10 size exclusion columns under strict anaerobic conditions.

For samples prepared without 2-mercaptoethanol, an additional 200 µL of HEPES buffer was added as a substitute. **Mq6 (Sec)** was reconstituted with 5 molar equiv. of dithiothreitol (DTT) *wrt* **Mq6 (Sec)**.

General Protocol for H_2-oxidation Experiments

Samples were handled in a Don Whitley A85 TG Anaerobic Workstation and analysed on a Shimadzu UV-2600 Spectrophotometer. The [FeFe]-hydrogenase used in these experiments was CaHydA from *Clostridium acetobutylicum*, obtained by recombinant overexpression and purification as previously described.[41,44] After reconstitution, [4Fe-4S]-cluster maquettes

were transferred into a quartz cuvette and sealed with a rubber turnover stopper. Samples were saturated with hydrogen by bubbling for 5 minutes. The reaction was started by adding an aliquot of CaHydA using a gastight syringe (Hamilton) from a 0.2 mg/mL stock to the desired enzyme concentration (in μg/mL). The final conc. of maquette peptide was 0.25 mM for all repeats. The reaction was monitored by UV-vis spectroscopy at room temperature.

Peptide Synthesis

Ac-KLCEGGCIACGACGGW-NH2 (FdM1)

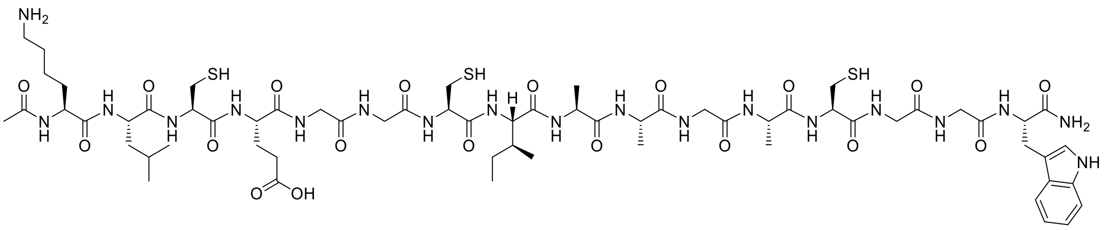

Peptide **FdM1** was synthesised using the general automated synthesiser protocol with microwave assistance on Rink Amide resin (0.2 mmol). The crude peptide was cleaved from the resin as described in the General Methods section, purified by preparative RP-HPLC (10-60% B over 30 min), and lyophilised to afford the desired peptide (**FdM1**, 249 mg, 0.162 mmol, 81% yield).

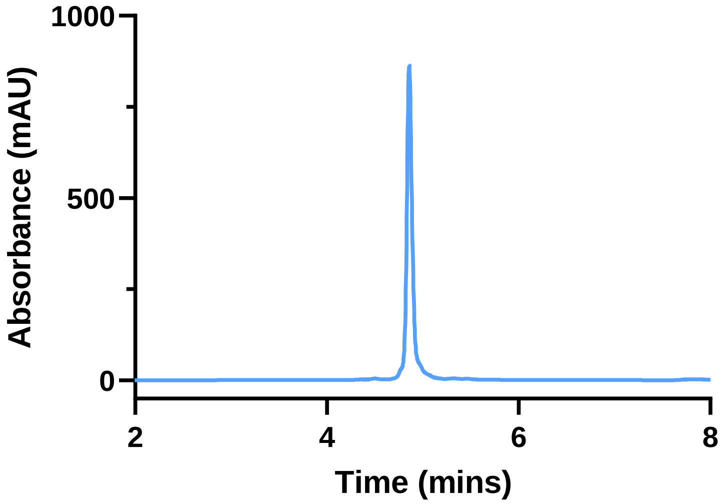

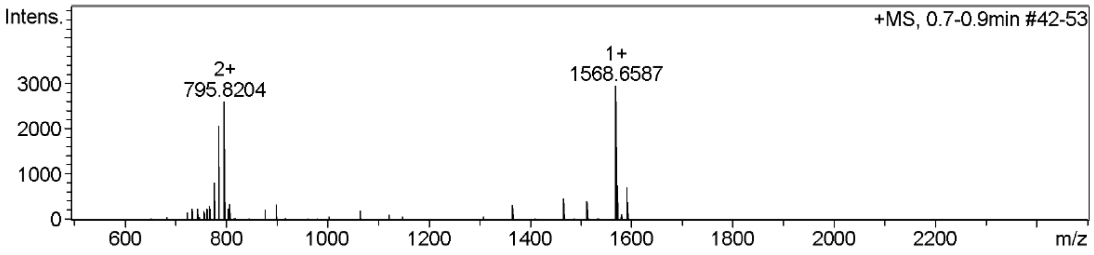

Figure S1 – Analytical HPLC trace and ESI MS of pure Ac-KLCEGGCIACGACGGW-NH2 (**FdM1**). Analytical gradient 10-60% B over 30 min, 210 nm. Calculated Mass $[M+Na]^+$: 1568.6523. Observed mass $[M+Na]^+$: 1568.6587.

Ac-KLGEGGGIAGGAGGGW-NH$_2$ (Mq2)

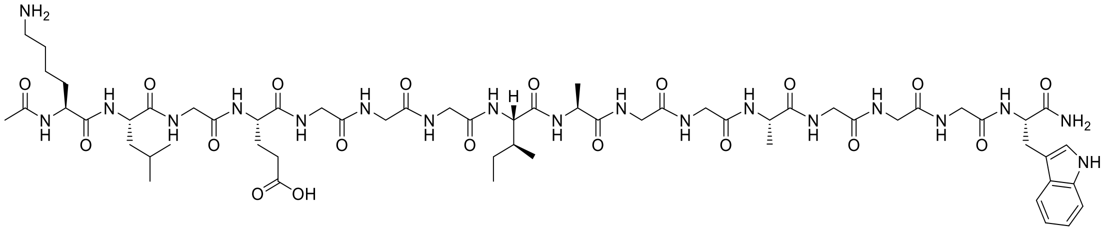

Peptide **Mq2** was synthesised using the general automated synthesiser protocol with microwave assistance on Rink Amide resin (0.2 mmol). The crude peptide was cleaved from the resin as described in the General Methods section, purified by preparative RP-HPLC (10-100% B over 30 min), and lyophilised to produce the desired peptide (**Mq2**, 150 mg, 0.110 mmol, 54% yield).

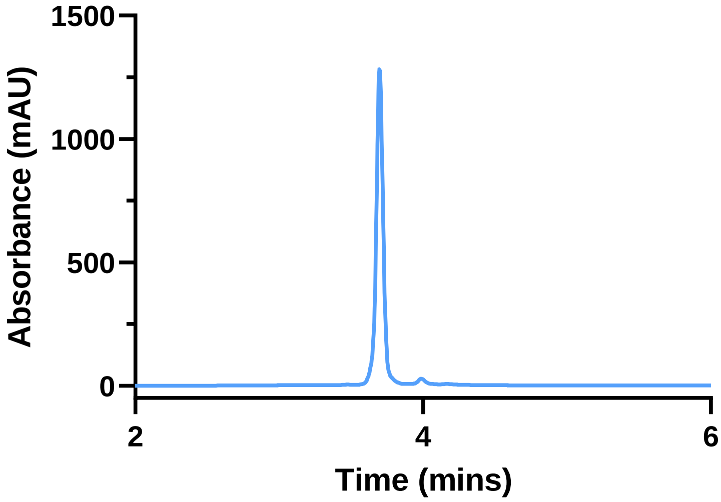

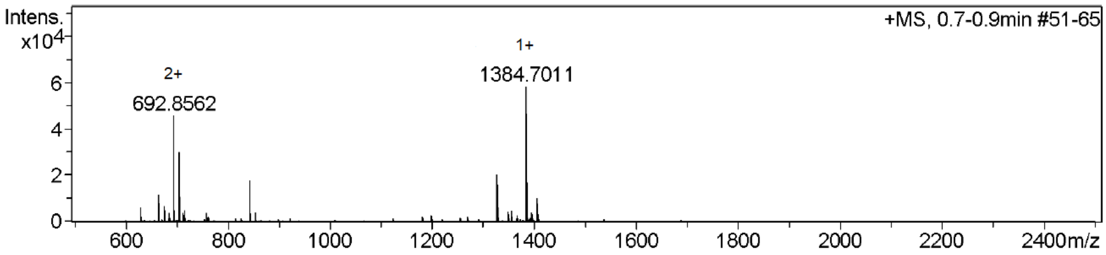

Figure S2 – Analytical HPLC trace and ESI MS of pure Ac-KLGEGGGIAGGAGGGW-NH$_2$ (**Mq2**). Analytical gradient 10-100% B over 5 min, 210 nm. Calculated Mass $[M+H]^+$: 1384.6735. Observed mass $[M+H]^+$: 1384.7011.

Ac-CGGGCGGCGGC-NH_2 (Mq3)

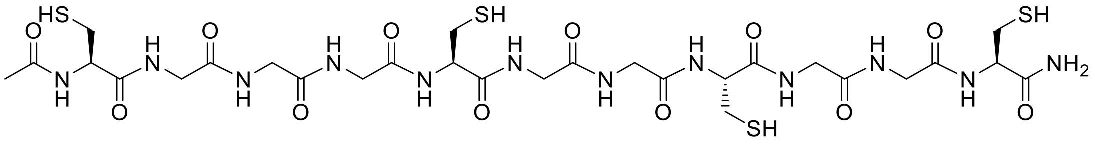

Peptide **Mq3** was synthesised using the general automated synthesiser protocol with microwave assistance on Rink Amide resin (0.2 mmol). The crude peptide was cleaved from the resin as described in the General Methods section, purified by preparative RP-HPLC (2-40% B over 30 min), and lyophilised to produce the desired peptide (**Mq3**, 106 mg, 0.120 mmol, 61% yield).

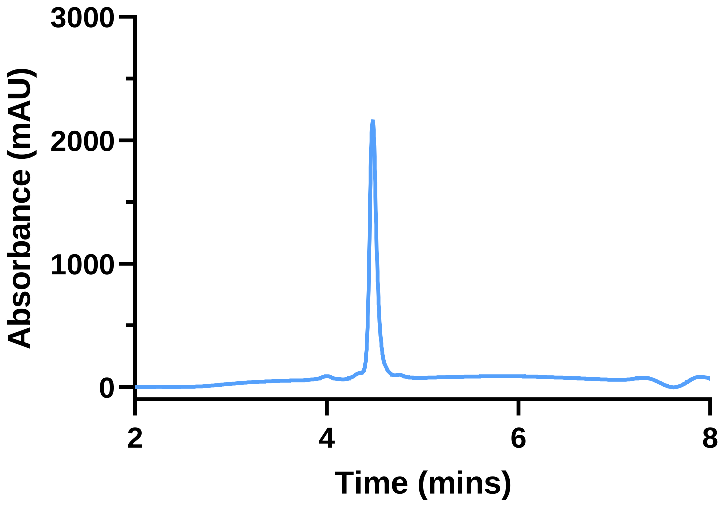

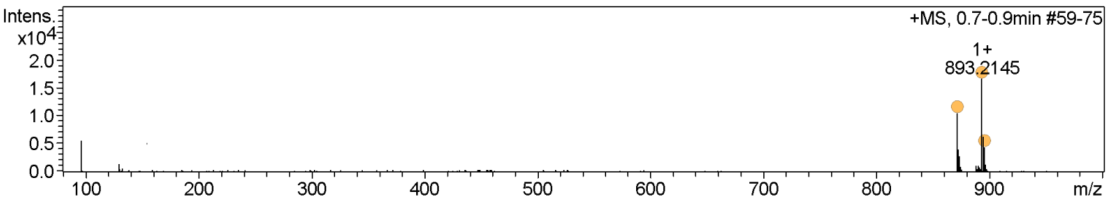

Figure S3 – Analytical HPLC trace and ESI MS of pure Ac-CGGGCGGCGGC-NH_2 (**Mq3**). Analytical gradient 2-40% B over 5 min, 210 nm. Calculated Mass $[M+Na]^+$: 894.9617. Observed mass $[M+Na]^+$: 893.2145.

Ac-GCGGGCGGCGGCG-NH2 (Mq4)

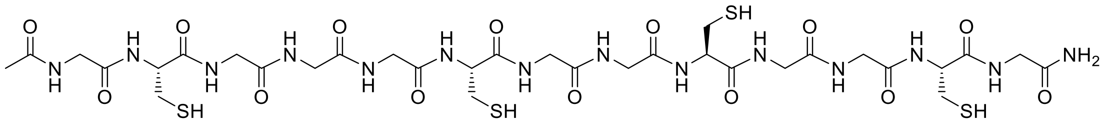

Peptide **Mq4** was synthesised using the general automated synthesiser protocol with microwave assistance on Rink Amide resin (0.2 mmol). The crude peptide was cleaved from the resin as described in the General Methods section, purified by preparative RP-HPLC (2-40% B over 30 min), and lyophilised to produce the desired peptide (**Mq4**, 85 mg, 0.086 mmol, 43% yield).

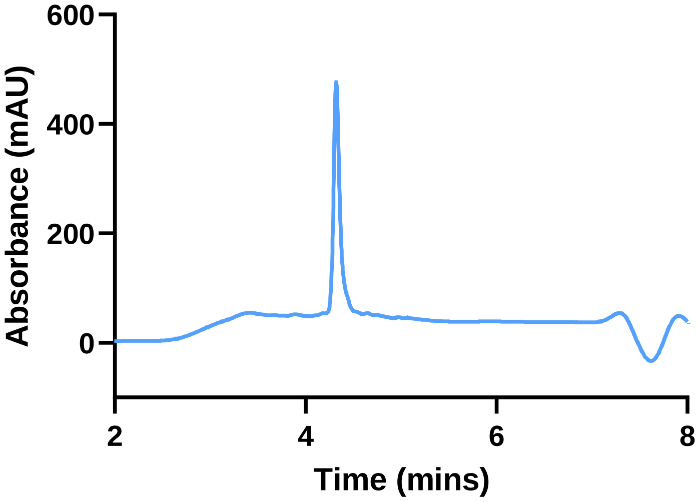

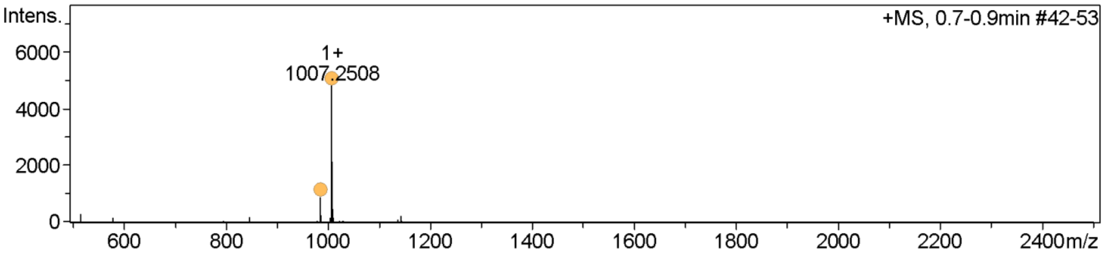

Figure S4 – Analytical HPLC trace and ESI MS of pure Ac-GCGGGCGGCGGCG-NH2 (**Mq4**). Analytical gradient 2-40% B over 5 min, 210 nm. Calculated Mass $[M+Na]^+$: 1008.2408. Observed mass $[M+Na]^+$: 1007.2508.

Ac-GCGGGCGGCGGCGY-NH2 (Mq5)

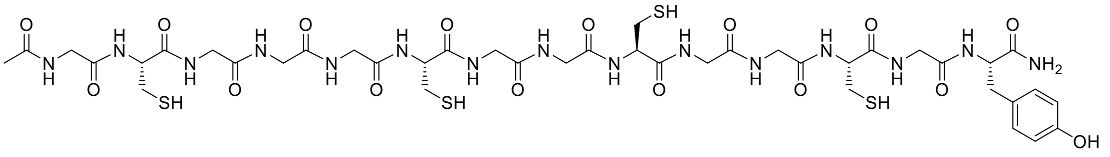

Peptide **Mq5** was synthesised using the general automated synthesiser protocol with microwave assistance on Rink Amide resin (0.2 mmol). The crude peptide was cleaved from the resin as described in the General Methods section, purified by preparative RP-HPLC (2-40% B over 30 min), and lyophilised to produce the desired peptide (**Mq5**, 165 mg, 0.140 mmol, 72% yield).

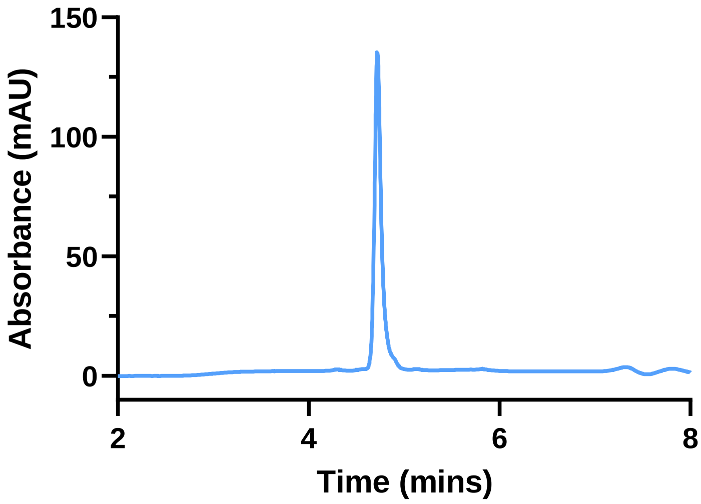

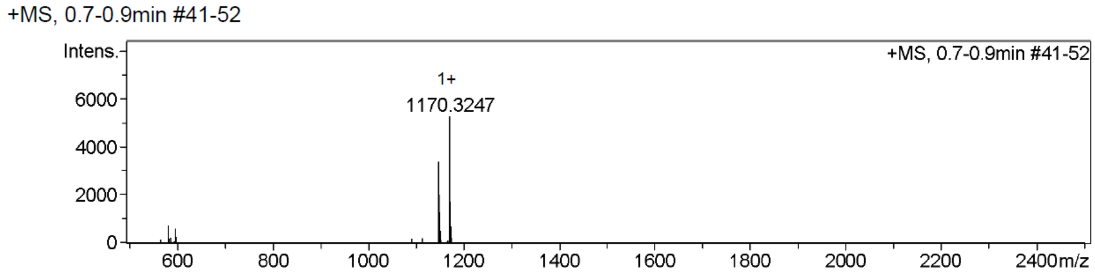

Figure S5 – Analytical HPLC trace and ESI MS of pure Ac-GCGGGCGGCGGCGY-NH2 (**Mq5**). Analytical gradient 2-40% B over 5 min, 210 nm. Calculated Mass $[M+Na]^+$: 1171.3041. Observed mass $[M+Na]^+$: 1170.3247.

Ac-GUGGGCGGCGGCGY-NH_2 (Mq6 (Sec))

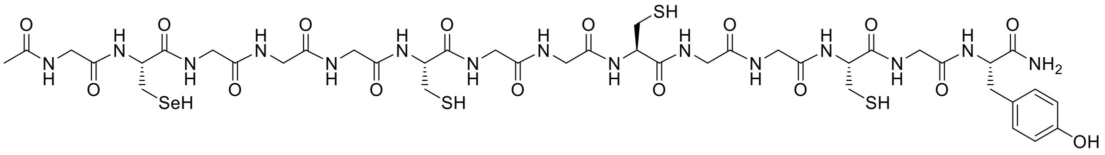

Peptide **Mq6 (Sec)** was synthesised using the general automated synthesiser protocol with microwave assistance on Rink Amide resin (0.2 mmol). Sec was incorporated as Fmoc-Sec(PMB)-OH (synthesised as previously described[47]) using standard coupling conditions. The crude peptide was cleaved from the resin as described in the General Methods section, purified by preparative RP-HPLC (2-95% B over 1 h), and lyophilised to produce the desired peptide (**Mq6 (Sec)**, 15 mg, 0.076 mmol, 38% yield).

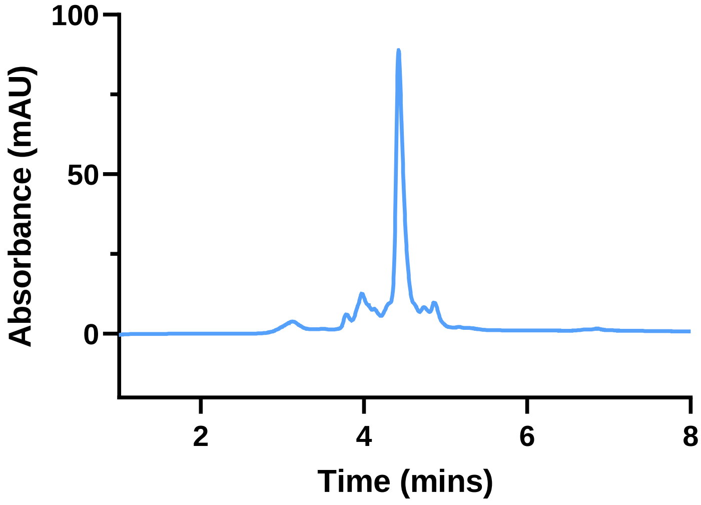

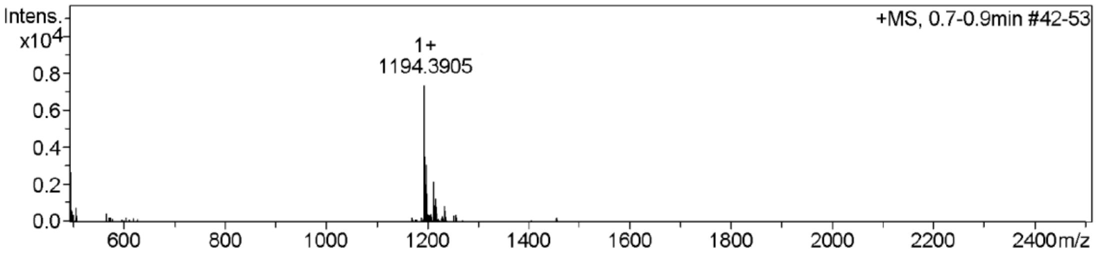

Figure S6 – Analytical HPLC trace and ESI MS of > 90% pure Ac-GUGGGCGGCGGCGY-NH_2 (**Mq6 (Sec)**). Analytical gradient 2-95% B over 10 min, 210 nm. Calculated Mass $[M+H]^+$: 1194.2596. Observed mass $[M+H]^+$: 1194.3905.

EPR data

See the General Methods section for the reconstitution procedure and details regarding EPR spectra acquisition.

FdM1-[4Fe4S]

Ac-KLCEGGCIACGACGGW-NH_2

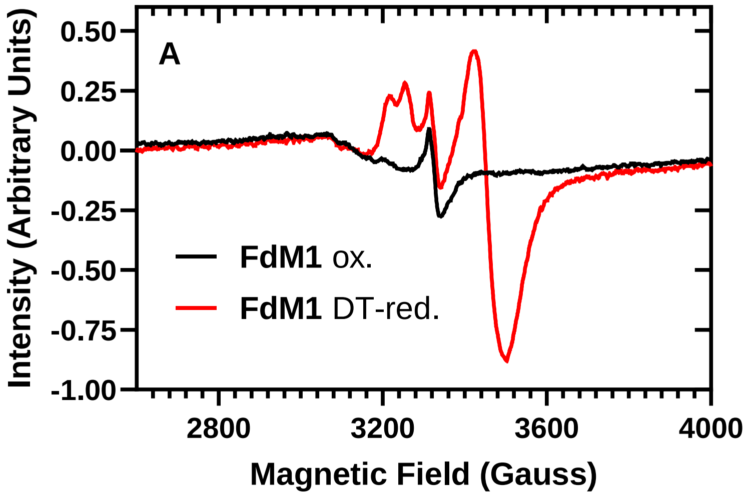

Figure S7 – X-band cw-EPR spectra at 20 K of a frozen solution of anaerobically reconstituted (in the presence of 2-mercaptoethanol, βME), oxidised **FdM1**-[4Fe4S] (cluster) (black trace) and dithionite-reduced **FdM1**-[4Fe4S] (red trace).

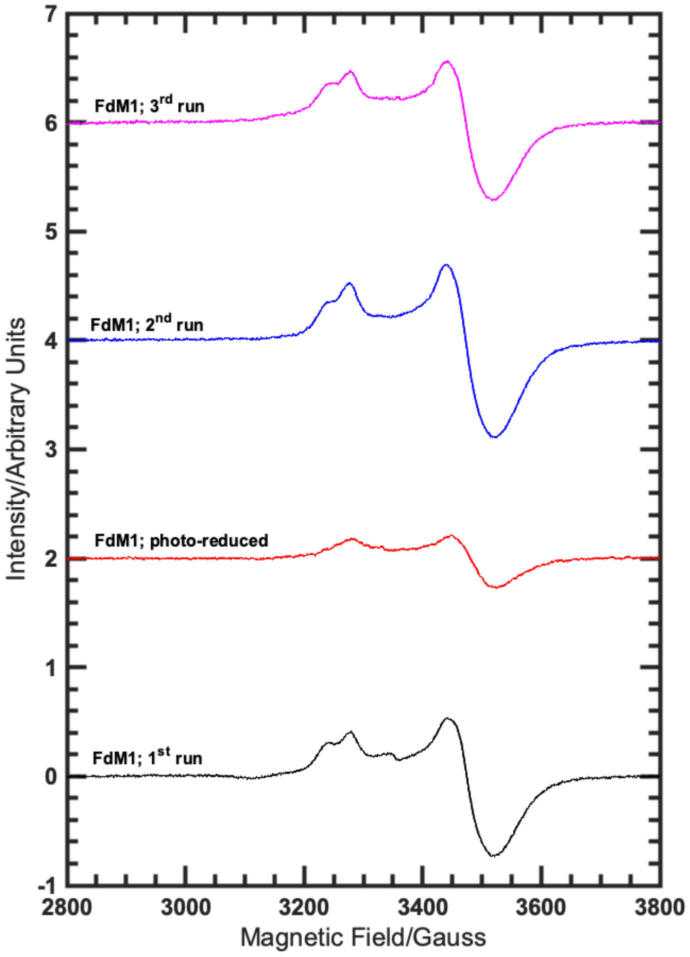

Figure S8 - X-band cw-EPR spectra at 20 K of a frozen solution of the anaerobically reconstituted, dithionite reduced, **FdM1** cluster measured at various time points. The **FdM1** sample labelled "photo-reduced" was reduced with NADH by the use of 365 nm LED. The doublet-structure observed between ~ 3200-3300 G implies the presence of two different conformations of the reduced $[4Fe4S]^+$ centre in the sample. *Experimental conditions*: microwave power 20 dB (2.19 mW), modulation amplitude 5 G, time constant 82 ms, conversion time 30 ms, sweep time 90 s, receiver gain 30 dB and an average microwave frequency of 9.385 GHz, T = 20 K.

EPR simulations

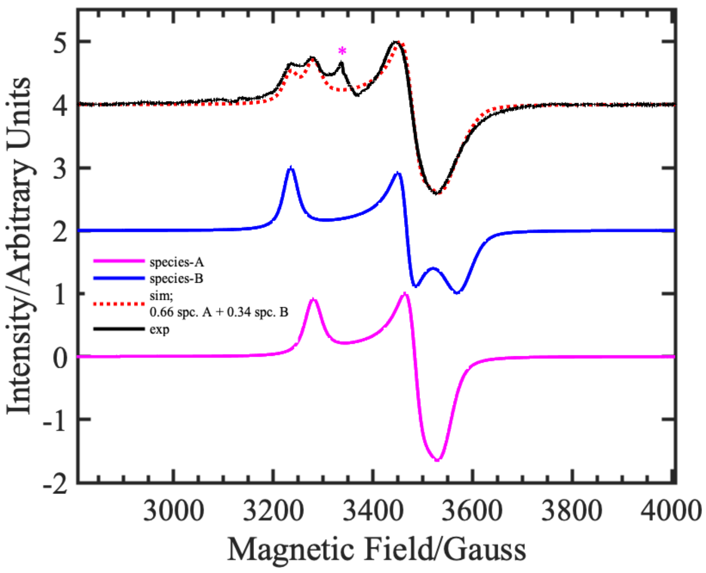

Figure S9 – X-band cw-EPR spectra of a frozen solution of anaerobically reconstituted, dithionite reduced, **FdM1** cluster measured at 20 K (black trace) and its simulations (red dotted trace with simulation parameters in Table 1 – main text). Magenta asterisk denotes the EPR signal arises from the buffer control.

The EPR spectrum was simulated (red dotted trace) by considering two, $S = ½$ species; the spin-Hamiltonian parameters used to model the experimental (black trace) spectrum are given below; the parameters are in good agreement with the reported values (see Table 1 and the experimental/simulated spectra[1]).

Species A (magenta trace); g = [1.896, 1.925, 2.046], line widths = [2.36 1.81] mT (milliTesla), H-strain = [43, 0 0], weight = 0.66.

Species B (blue trace); g = [1.868, 1.935, 2.071], line widths = [2.07 1.0] mT (milliTesla), H-strain = [77, 0 0], weight = 0.34.

The EPR signals indicated by the magenta asterisk mark are from the buffer-control.

FdM1 reconstitution without βME

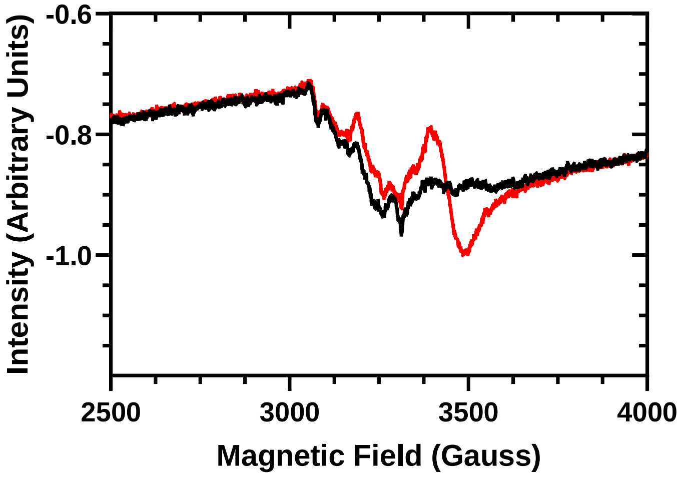

Figure S10 - X-band cw-EPR spectra at 20 K of a frozen solution of anaerobically reconstituted (in the absence of 2-mercaptoethanol, βME), oxidised **FdM1** cluster (black trace) and dithionite-reduced **FdM1** cluster (red trace).

Mq3-[Fe_4S_4]

Ac-CGGGCGGCGGC-NH_2

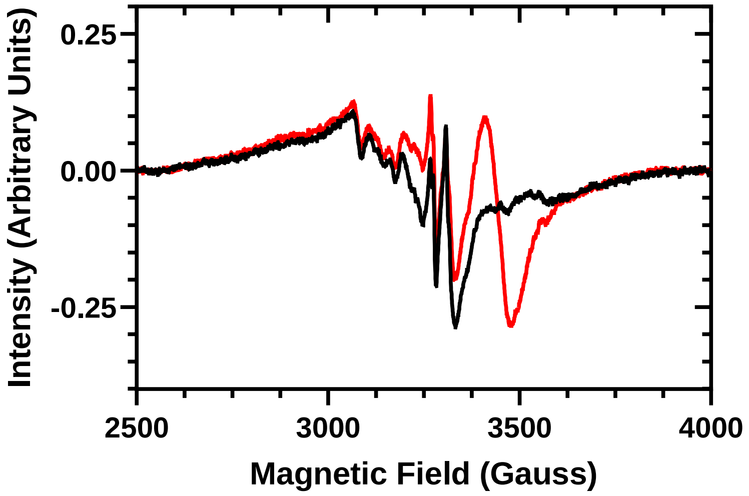

Figure S11 - X-band cw-EPR spectra at 20 K of a frozen solution of anaerobically reconstituted (in the absence of 2-mercaptoethanol, βME), oxidised **Mq3** cluster (black trace) and dithionite-reduced **Mq3** cluster (red trace).

Mq4-[Fe_4S_4]

Ac-GCGGGCGGCGGCG-NH_2

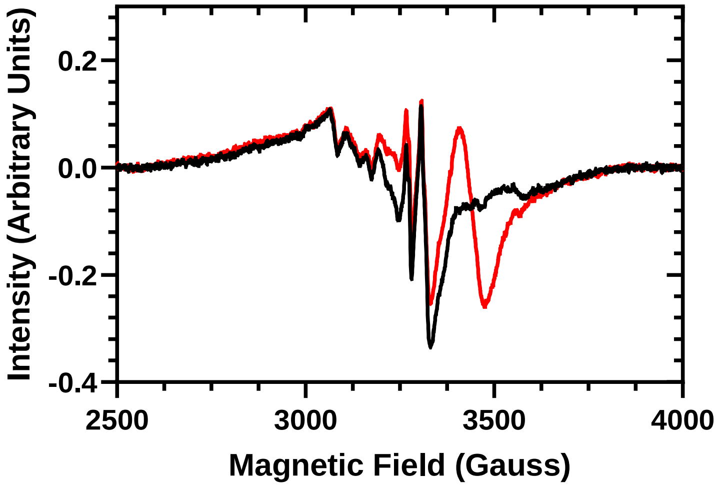

Figure S12 - X-band cw-EPR spectra at 20 K of a frozen solution of anaerobically reconstituted (in the absence of 2-mercaptoethanol, βME), oxidised **Mq4** cluster (black trace) and dithionite-reduced **Mq4** cluster (red trace).

Mq5-[Fe_4S_4]

Ac-GCGGGCGGCGGCGY-NH_2

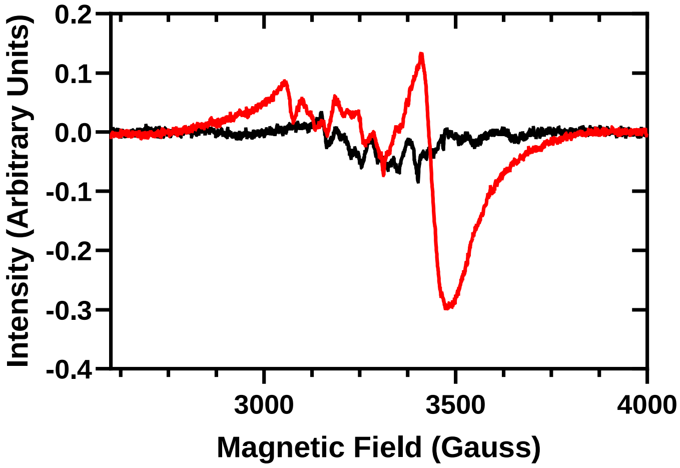

Figure S13 - X-band cw-EPR spectra at 20 K of a frozen solution of anaerobically reconstituted (in the absence of 2-mercaptoethanol, βME), oxidised **Mq5** cluster (black trace) and dithionite-reduced **Mq5** cluster (red trace).

Mq6 (Sec)-[Fe_4S_4]

Ac-GUGGGCGGCGGCGY-NH_2

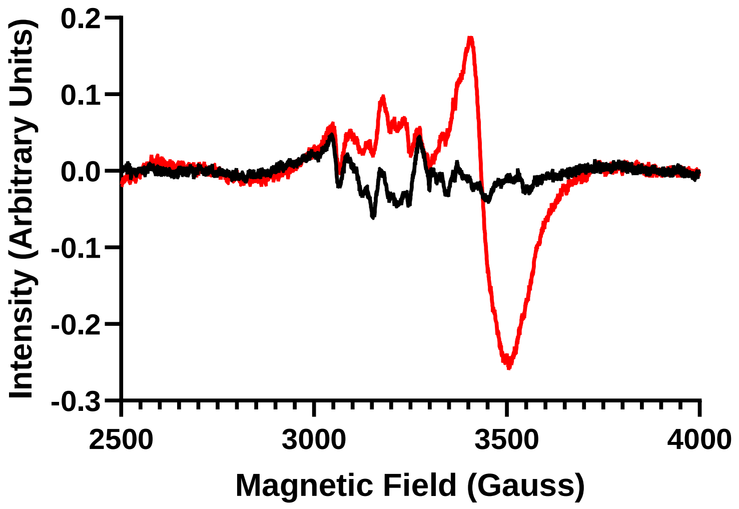

Figure S14 – X-band cw-EPR spectra at 20 K of a frozen solution of anaerobically reconstituted (in the presence of dithiothreitol, DTT), oxidised **Mq6 (Sec)** cluster (black trace) and dithionite-reduced **Mq6 (Sec)** cluster.

Estimation of $[4Fe\text{-}4S]^{+}$ Formation Relative to FdM1

Although several attempts were made to reconstitute the $[4Fe\text{-}4S]^{2+}$ cluster into the various maquette peptides without trace amount of Mn(II) ion impurities, it was unavoidable. The EPR spectra of all the oxidized maquette peptides show the presence of EPR signals arising from the Mn(II) ion which overlap with the EPR signals from the one-electron reduced, $[4Fe\text{-}4S]^{+}$ cluster, when the oxidized maquette sample was chemically reduced with the dithionite solution or by NADH photo-reduction. In addition, the EPR spectra also contain a sharp EPR signal arising from an unknown radical signal (likely from HEPES buffer; Figure S7) along with weak EPR cavity background signal. These signals showed change in intensity after the samples' reduction/reaction with the dithionite solution. This made the quantification process a challenging one. In order to overcome this issue and minimise the error due to the background and signals form impurities, the spectrum of the oxidized maquette sample was subtracted from that of the corresponding reduced maquette sample. Although, this process has removed majority of the background/impurity signals, the subtraction was not perfect and this imperfection leads to distortion of the shape of the EPR signals, especially at the "high-g" region (close to free-electron g-value). It is also noteworthy that all the spectra displayed in the main text have been field-corrected (relative to that of 9.382362 GHz - frequency) due to slight differences in the observed microwave frequencies between different experiments. Further, field-correction was applied by measuring a standard sample, strong-pitch under the identical experimental conditions, so the extracted "g-matrix" is accurate. It is also understood that large "error" in the spin-quantification is likely due to "poor" baseline, so additional care was taken when working with the first-derivative and first-integral spectra of each sample. To avoid over-estimation and be on the safer side, the estimated error in the spin-quantification and/or $[4Fe\text{-}4S]^{+}$ cluster formation is "20±5%", given the number of manipulations that each spectrum has gone through. This assumption is equally applicable for the extracted "line widths" and the "g_3/g_{max}" of the simulated spectra. The original data before they have gone through any manipulation are given in Figures S15 and S16.

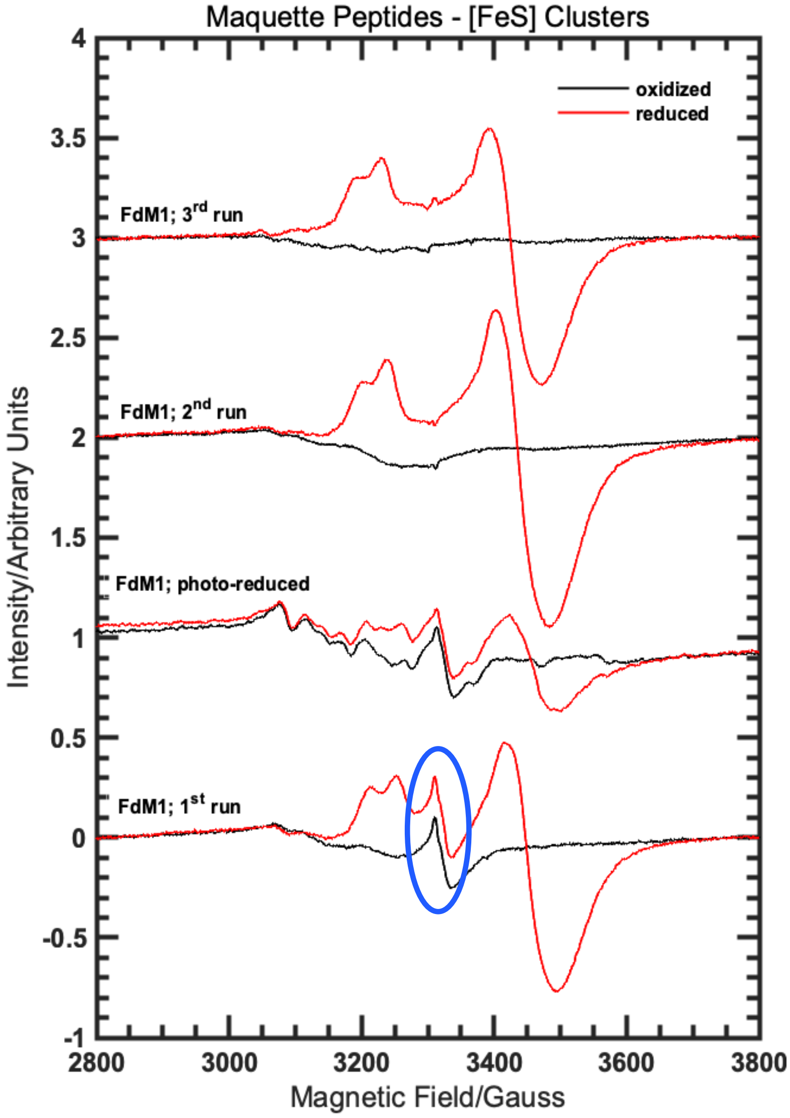

Figure S15. X-band cw-EPR spectra at 20 K of a frozen solution of **FdM1** peptide prepared and measured at various time points. The black and red traces are before and after reduction of the reconstituted, anaerobic **FdM1** peptide with dithionite. The **FdM1** sample labelled "photo-reduced" was reduced with NADH by the use of 365 nm LED. The EPR signals observed in the black traces are from the Mn(II) impurity, plus a sharp, unknown radical like signal as indicated by the blue ellipse (~ 3330 G). Experimental *Conditions*; MW power 20 dB, MA 5G, time constant 82 ms, conversion time 30 ms, sweep time 90 s, receiver gain 30 dB, average microwave frequency 9.385 GHz, temperature 20 K.

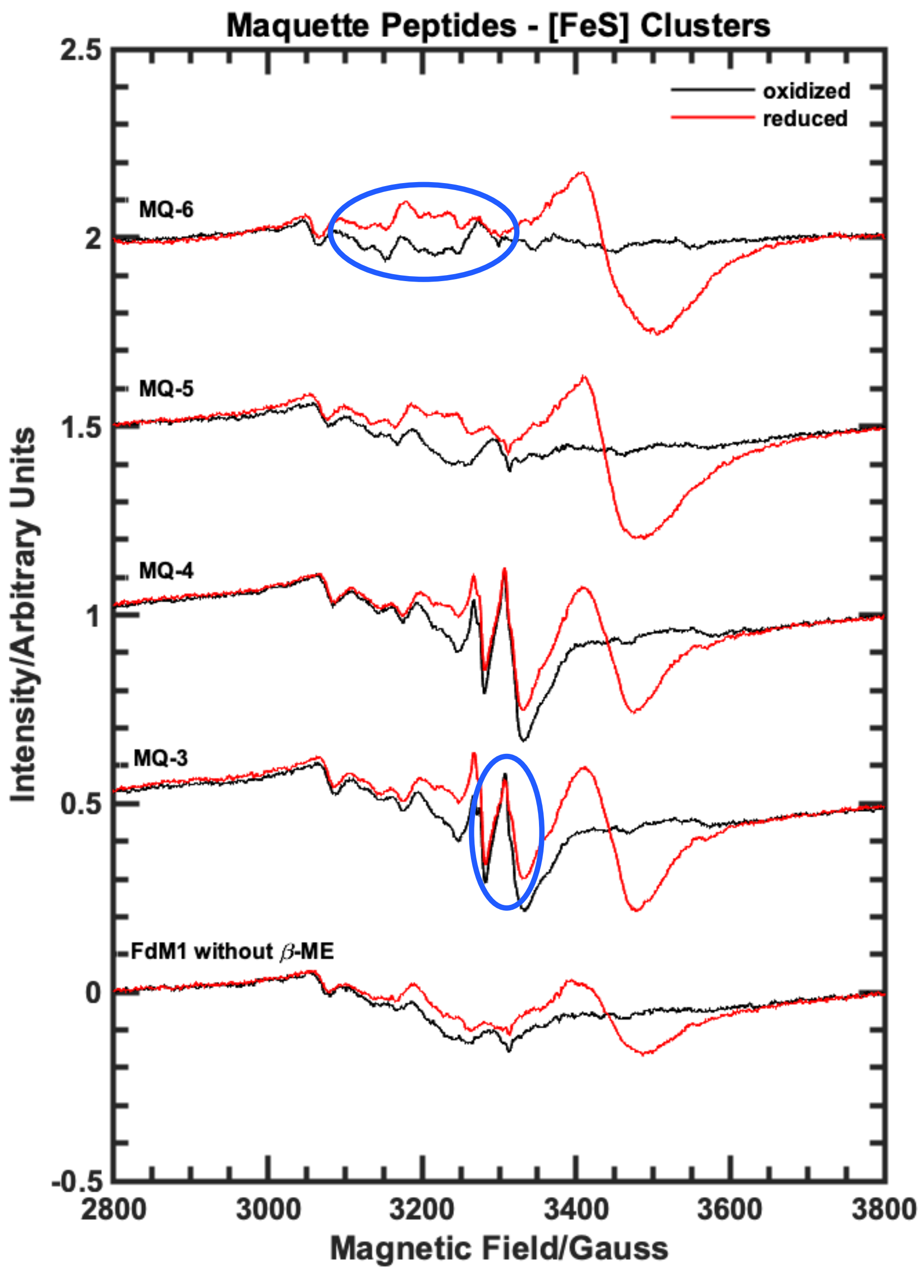

Figure S16. X-band cw-EPR spectra at 20 K of a frozen solution of various maquettes measured at 20 K. The blue ellipses imply that the EPR signals observed in the black traces change in intensity after reaction/reduction with dithionite. The black and red traces are before and after reduction of the reconstituted, anaerobic maquettes with dithionite.

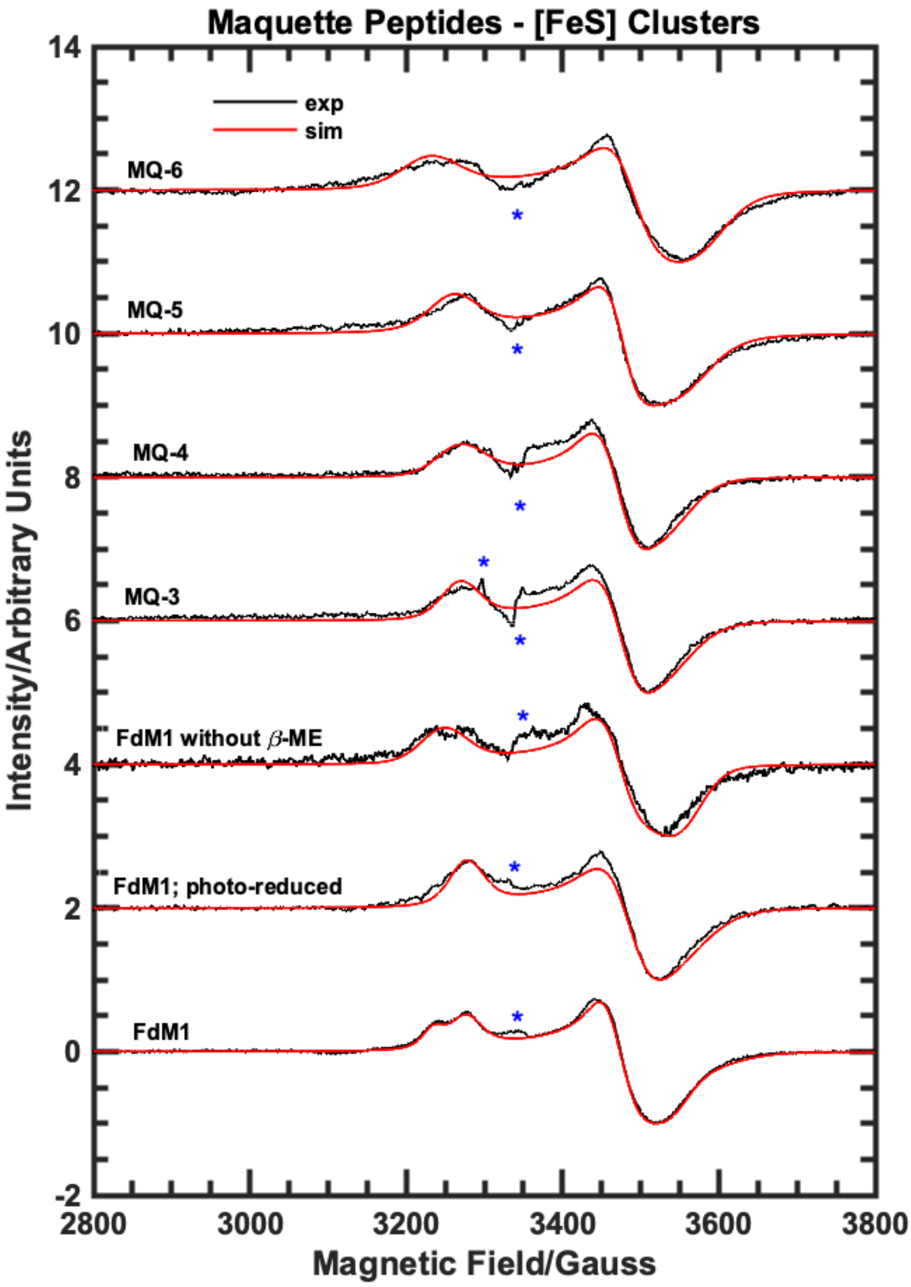

Figure S17. Experimental and simulated cw-EPR spectra of various maquettes measured at 20 K. The blue asterisk marks imply that these signals are due to imperfect background subtraction. This also plausibly accounts for the observed differences in intensity of the simulated spectra, especially the EPR signal at the magnetic field, ~ 3500 G. The spin-Hamiltonian parameters used to model the experimental data are provided in **Table 1**.

Table S1. Quantification of formation of one-electron reduced, $[4Fe\text{-}4S]^{+1}$ cluster in the various maquette peptides. The estimated error for formation of the cluster relative to FdM1 is 20±5% as detailed above.

Sample	Double Integral	Relative Percentage[a] *wrt* 6.0908×10^4
FdM1 – 1st run	5.4042×10^4	100 %
FdM1 – photo-reduced	2.0738×10^4	34 %
FdM1 – 2nd run	7.0057×10^4	100 %
FdM1 – 3rd run	5.8625×10^4	100 %
FdM1-without β-ME	1.0289×10^4	17 %
MQ3	1.2812×10^4	21 %
MQ4	1.1643×10^4	19 %
MQ5	2.4192×10^4	40 %
MQ6	2.8107×10^4	46 %

[a] = average of the double integral of the **FdM1** runs (6.0908×10^4); the quantification/formation of other Mq's was estimated against the average double integral obtained for the **FdM1** peptide. The estimated error on the formation of the [Fe-S] cluster relative to that of **FdM1** peptide is 20±5% due to the reasons provided under "double integral" heading.

The quantification of the formation of one-electron reduced, $[4Fe\text{-}4S]^{+1}$ cluster in the various maquette peptides was compared against the **FdM1** peptide cluster. To minimize the quantification error, the EPR spectrum of the **FdM1**-peptide was collected at three different time points/periods as **FdM1**-1st run, **FdM1**-2nd run and **FdM1**-3rd run respectively. The double integral of each run is considered as "100 %" and the average (6.0908×10^4) is taken for quantification of the formation of one-electron reduced, $[4Fe\text{-}4S]^{+1}$ cluster in the other maquette peptides.

Cyclic Voltammetry of Peptide-$[4Fe4S]^{2+/+}$ Clusters

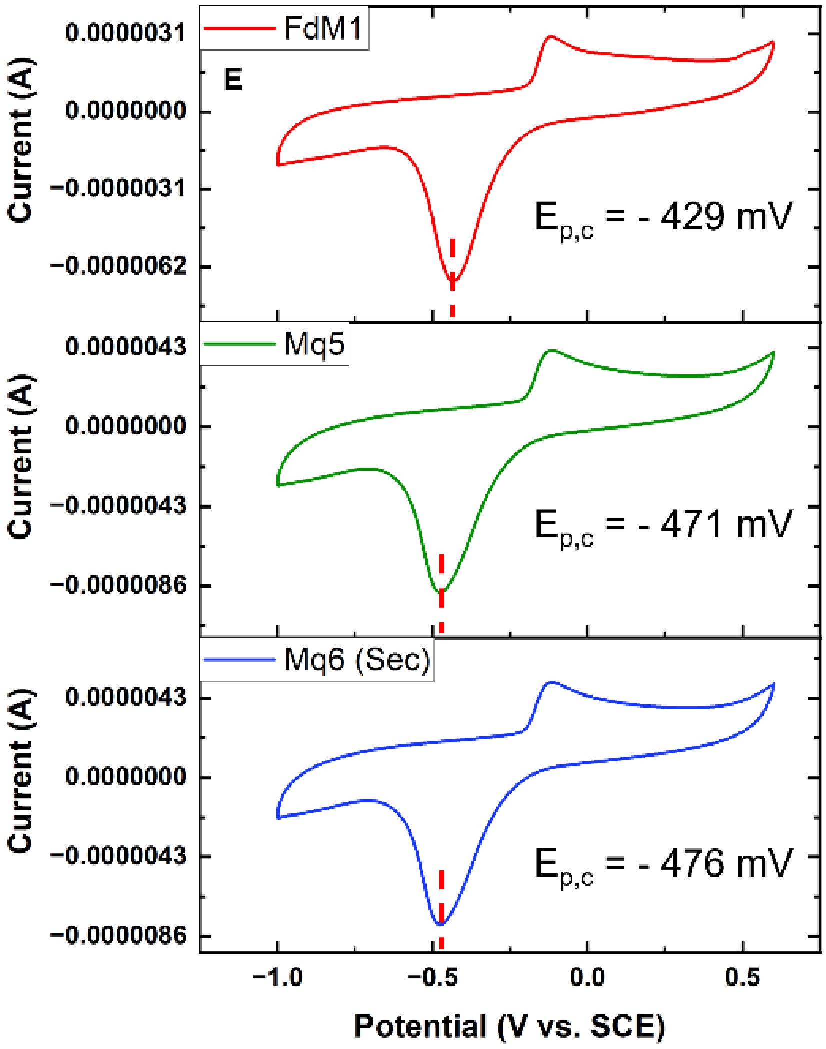

Figure S18 – Cyclic Voltammetry (CV) analysis (average of 10 cycles) of **FdM1, Mq5,** and **Mq6 (Sec)** anaerobically reconstituted in 50 mM HEPES, 10 mM KCl, 100 mM NaCl, pH 8.0. **FdM1** and **Mq5** were reconstituted in the absence of βME; **Mq6 (Sec)** was reconstituted with 5 equiv. of dithiolthreitol (DTT).

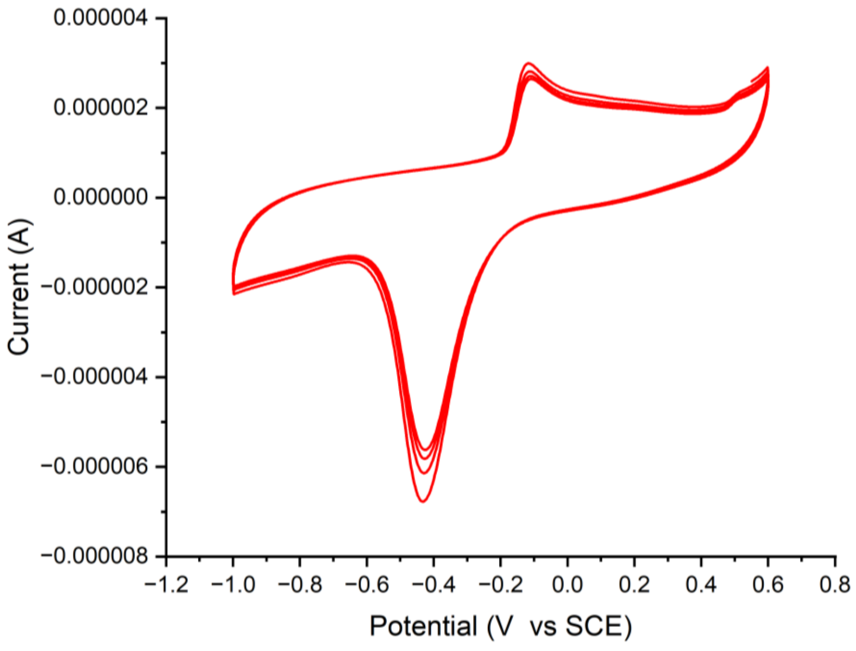

Figure S19 – Cyclic Voltammetry (CV) trace for **FdM1** vs saturated calomel electrode (SCE). $[4Fe4S]^{2+/+}$ cluster, reconstituted anaerobically in the absence of βME.

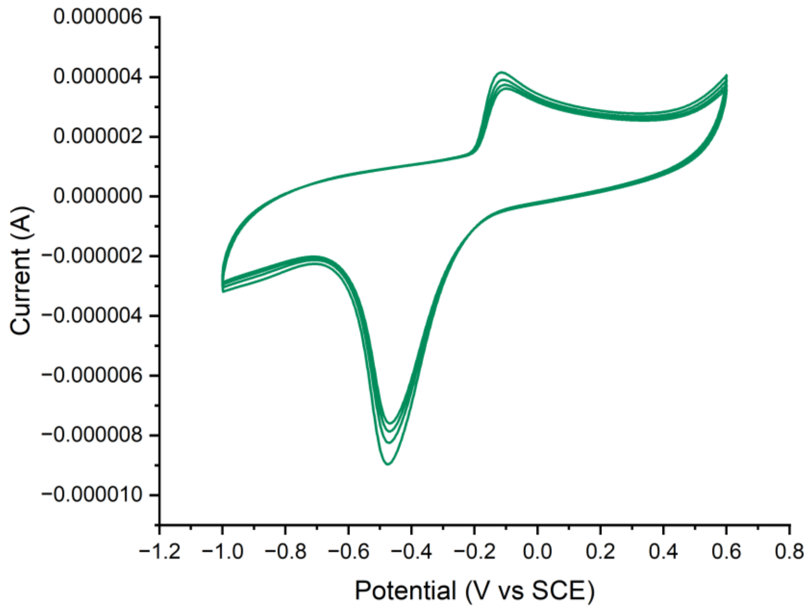

Figure S20 – Cyclic Voltammetry (CV) trace for **Mq5** vs saturated calomel electrode (SCE). $[4Fe4S]^{2+/+}$ cluster, reconstituted anaerobically in the absence of βME.

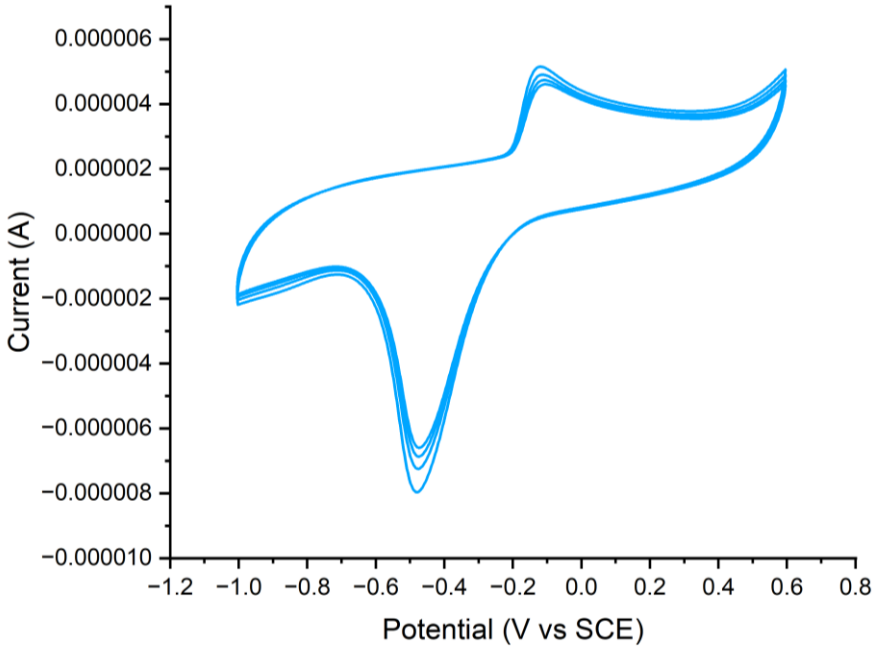

Figure S21 – Cyclic Voltammetry (CV) trace for **Mq6 (Sec)** vs saturated calomel electrode (SCE). $[4Fe4S]^{2+/+}$ cluster, reconstituted anaerobically with 5 equiv. of DTT.

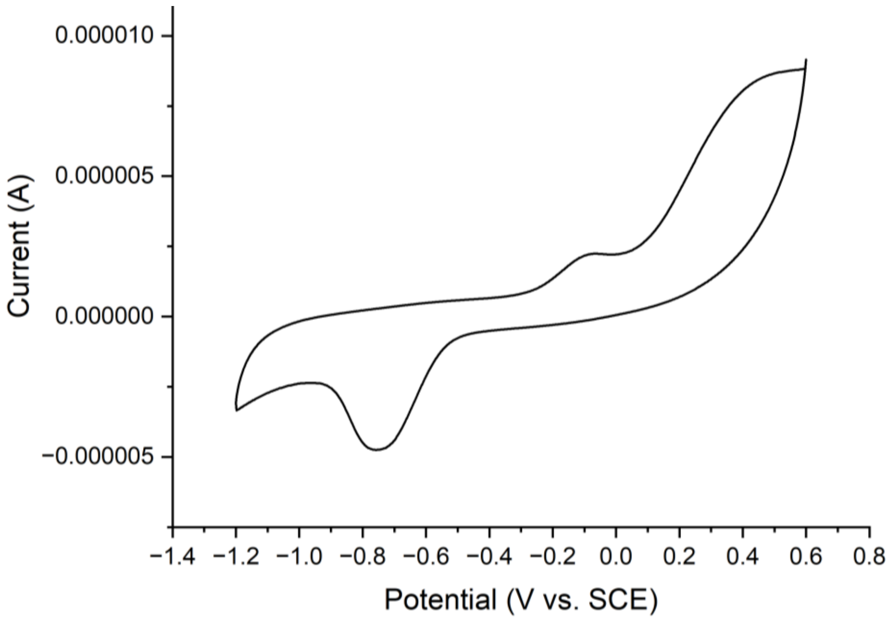

Figure S22 – Cyclic Voltammetry (CV) trace for Na_2S (3 mM) vs saturated calomel electrode (SCE)

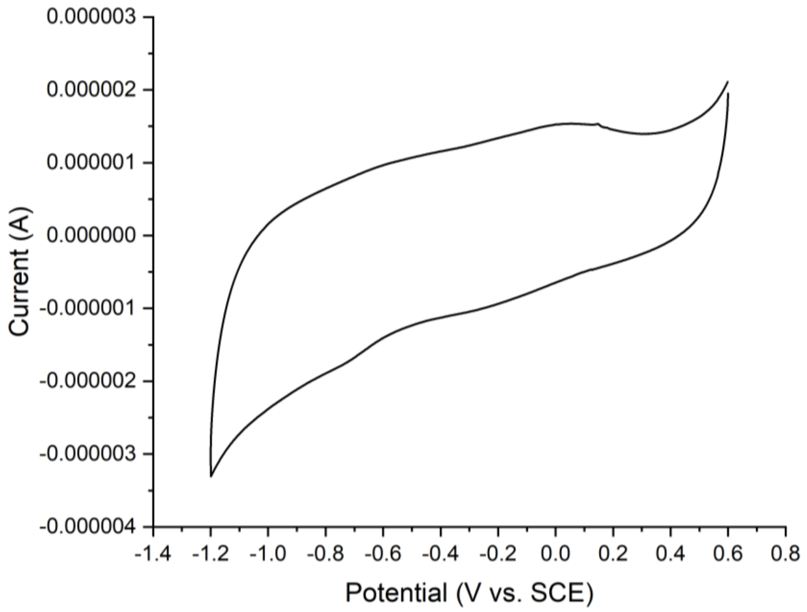

Figure S23 – Cyclic Voltammetry (CV) trace for peptide **FdM1** (0.5 mM) vs saturated calomel electrode (SCE)

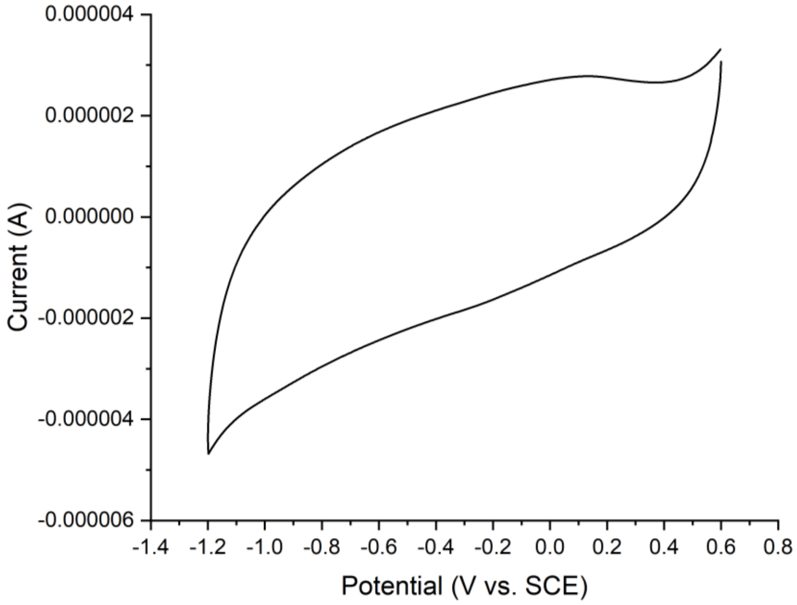

Figure S24 – Cyclic Voltammetry (CV) trace for peptide **Mq5** (0.5 mM) vs saturated calomel electrode (SCE)

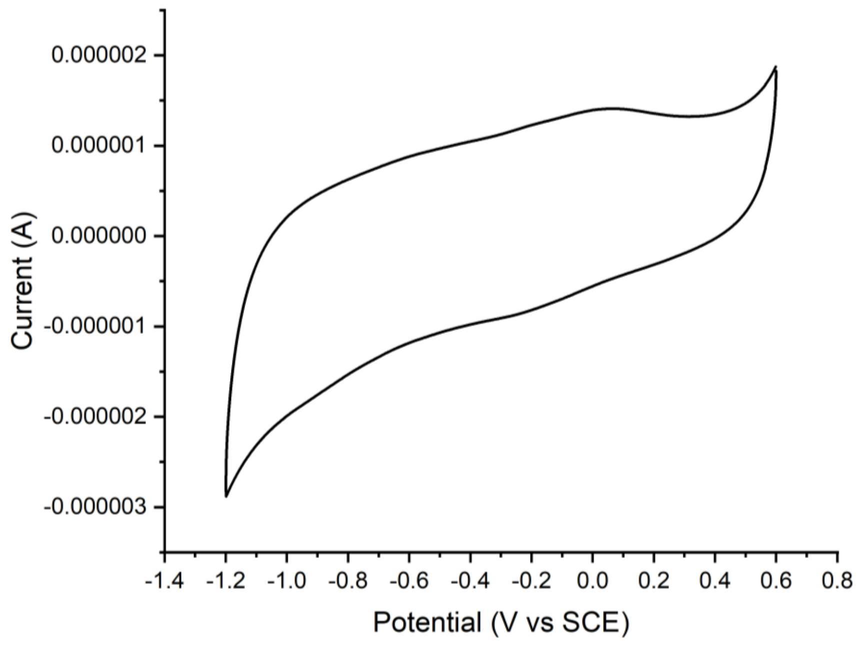

Figure S25 – Cyclic Voltammetry (CV) trace for aerobically-reconstituted **FdM1** vs saturated calomel electrode (SCE).

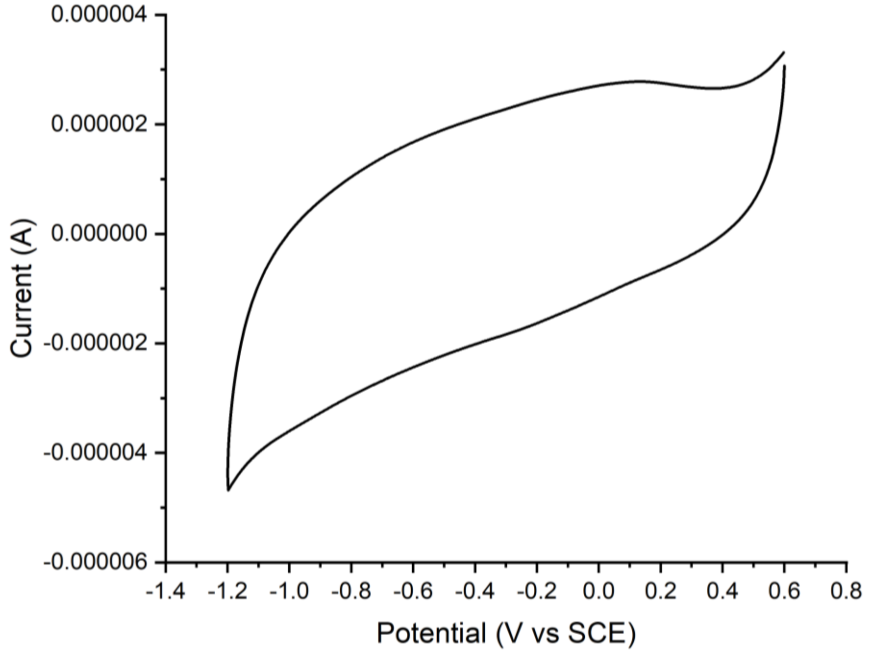

Figure S26 – Cyclic Voltammetry (CV) trace for aerobically-reconstituted **Mq5** vs saturated calomel electrode (SCE).

UV-Vis Analysis of Peptide-[4Fe4S]$^{2+/+}$ Clusters

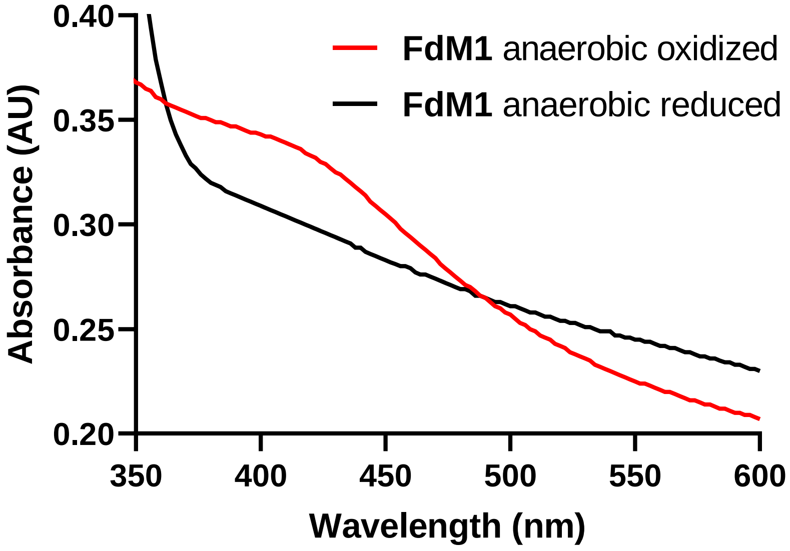

Figure S27 – UV-Vis analysis of oxidised (red trace) and dithionite-reduced (black trace) **FdM1** cluster reconstituted in the absence of βME under anaerobic conditions.

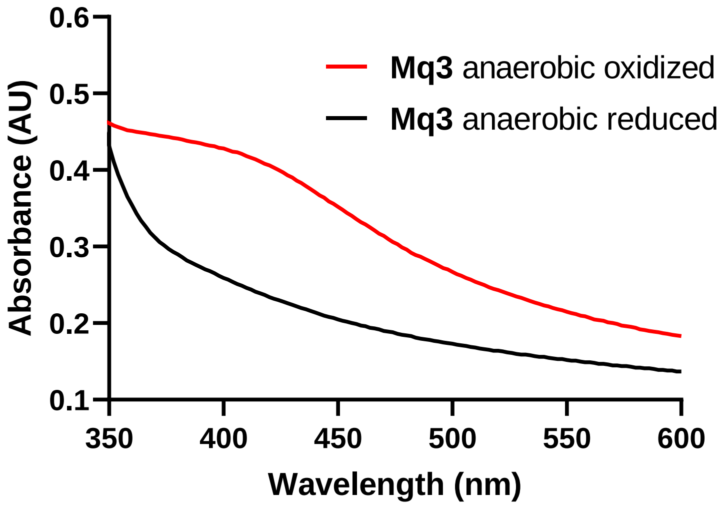

Figure S28 – UV-Vis analysis of oxidised (red trace) and dithionite-reduced (black trace) **Mq3** cluster reconstituted in the absence of βME under anaerobic conditions.

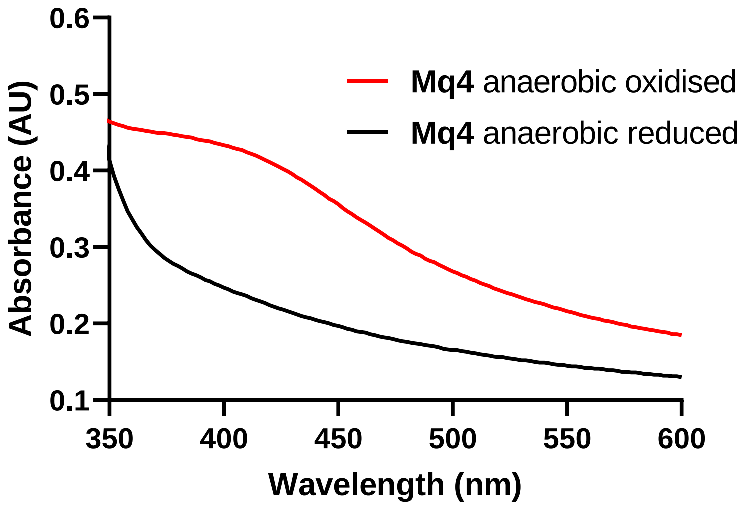

Figure S29 – UV-Vis analysis of oxidised (red trace) and dithionite-reduced (black trace) **Mq4** cluster reconstituted in the absence of βME under anaerobic conditions.

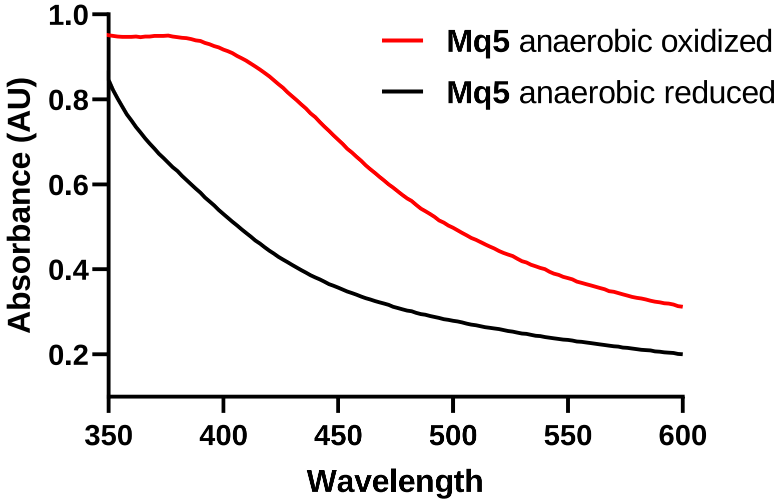

Figure S30 – UV-Vis analysis of oxidised (red trace) and dithionite-reduced (black trace) **Mq5** cluster reconstituted in the absence of βME under anaerobic conditions.

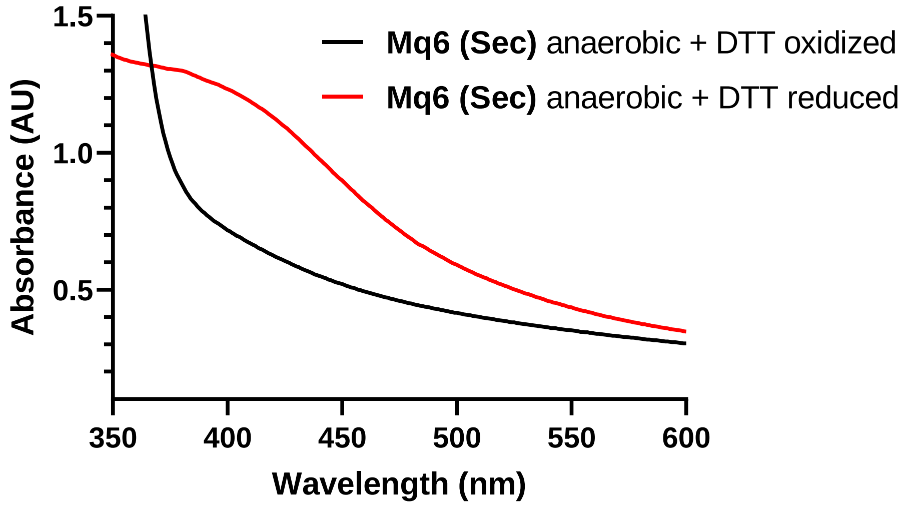

Figure S31 – UV-Vis analysis of oxidised (red trace) and dithionite-reduced (black trace) **Mq6 (Sec)** cluster reconstituted with DTT under anaerobic conditions.

FdM1 Maquette Integrated into a Hydrogenase Mediated H_2/H^+ Redox Couple

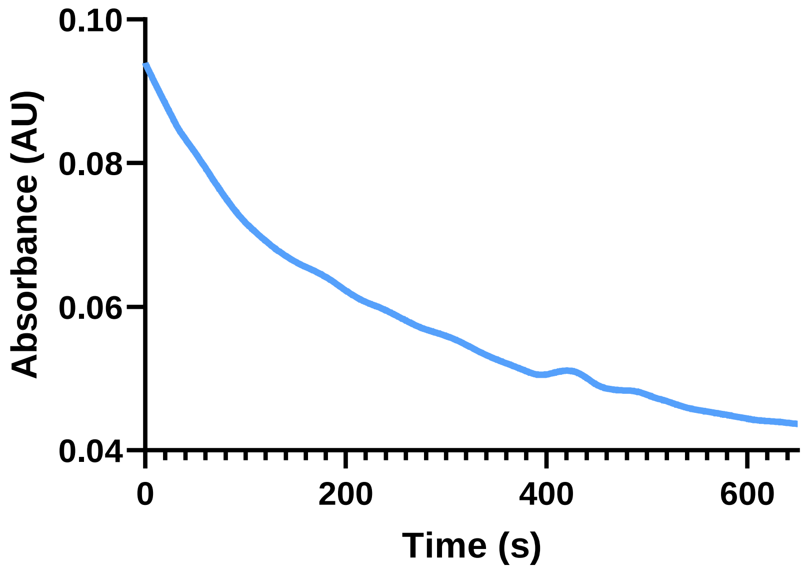

Figure S32 – Decrease in absorbance at 420 nm monitored after addition of CaHydA to the **FdM1** maquette cluster sparged with H_2.

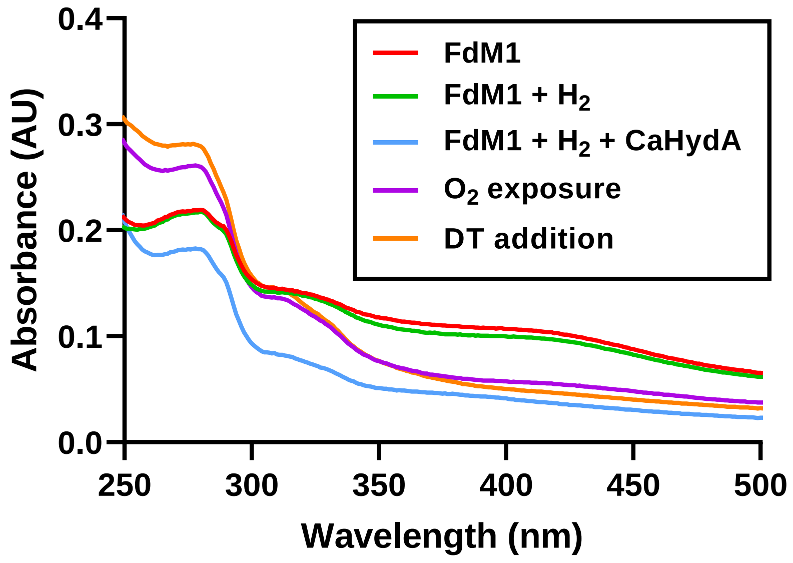

Figure S33 – UV-Vis analysis of the **FdM1** maquette cluster before and after sparging with H_2, after CaHydA addition, O_2 exposure, and DT reduction. Abs. at 420 nm represents the LMCT band for the cluster.

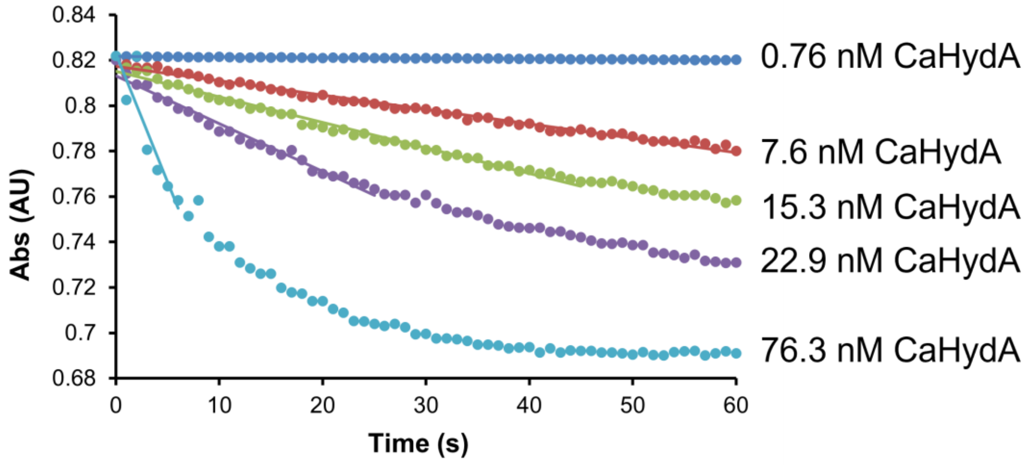

Figure S34 – Rate plot for CaHydA-mediated H_2-oxidation with trend-line fit for initial slopes shown. Data generated by monitoring the absorbance decrease at 420 nm due to **[4Fe4S]-Mq5** reduction.

References

[17] S. E. Mulholland, B. R. Gibney, F. Rabanal, P. L. Dutton, *J. Am. Chem. Soc.* **1998**, *120*, 10296-10302.

[23] A. Galambas, J. Miller, M. Jones, E. McDaniel, M. Lukes, H. Watts, V. Copié, J. B. Broderick, R. K. Szilagyi, E. M. Shepard, *J. Biol. Inorg. Chem.* **2019**, *24*, 793-807.

[41] P. W. King, M. C. Posewitz, M. L. Ghirardi, M. Seibert, *J. Bacteriol.* **2006**, *188*, 2163-2172.

[44] S. Morra, A. Cordara, G. Gilardi, F. Valetti, *Protein Sci.* **2015**, *24*, 2090-2094.

[45] S. S. Eaton, T. Ngendahimana, G. R. Eaton, A. R. Jupp, D. W. Stephan, *J. Magn. Reson.* **2018**, *290*, 76-84.

[46] S. Stoll, A. Schweiger, *J. Magn. Reson.* **2006**, *178*, 42-55.

[47] M. D. Gieselman, L. Xie, W. A. Van Der Donk, *Org. Lett.* **2001**, *3*, 1331-1334.
